# The impact of heat therapy on neuromuscular function and muscle atrophy in diabetic rats

**DOI:** 10.3389/fphys.2022.1039588

**Published:** 2023-01-05

**Authors:** Abdullah T. AlSabagh, Muddanna S. Rao, Waleed M. Renno

**Affiliations:** Department of Anatomy, College of Medicine, Health Sciences Center, Kuwait University, Kuwait city, Kuwait

**Keywords:** atrophy, diabetes, heat therapy, hypertrophy, skeletal muscle, degeneration markers, regeneration markers

## Abstract

**Introduction:** Diabetes Mellitus (DM) is the most common metabolic disease worldwide and is associated with many systemic complications. Muscle atrophy is one of the significant complications in DM patients, making routine tasks laborious as atrophy continues. It is known that heat stress stimulates heat shock proteins and other proteins that maintain muscle mass; however, it is not thoroughly studied in diabetic conditions. This study addressed whether heat therapy can attenuate muscle atrophy in STZ-induced diabetic rats and explored its mechanism of action on specific muscle proteins.

**Methods:** Male Sprague Dawley rats were randomly divided into short-term (3 weeks) and long-term (6 weeks) experiments. In each experiment rats were divided into control, heat therapy, diabetic and diabetic + heat therapy groups. Rats in heat therapy groups were exposed to heat therapy for 30 min daily for three or six weeks in a temperature-controlled (42°C) chamber.

**Results:** The attenuation of neuromuscular functions assessed by Rotarod, Kondziella’s inverted screen, and extensor postural thrust tests showed that diabetic rats exposed to heat therapy performed significantly better than diabetic controls. Muscle cross sectional area data established that heat therapy reduced muscle atrophy by 34.3% within 3 weeks and 44.1% within 6 weeks in the diabetic groups. Further, heat therapy significantly decreased muscle atrophy markers (CD68, KLF, and MAFbx) and significantly elevated muscle hypertrophy markers (AKT, mTOR, and HSP70).

**Conclusions:** This study shows the relevance and clinical significance of utilizing heat therapy as a viable treatment to attenuate muscle atrophy in diabetic patients.

## 1. Introduction

More than 420 million people suffer worldwide from diabetes mellitus (DM), making it one of the most prevalent diseases in the world. DM is a chronic metabolic disease characterized by increased blood glucose levels, mainly due to insulin deficiency or the inability of the cells to utilize insulin for glucose metabolism (Izzo et al., 2021). Diabetes may lead to several health complications in the heart, eyes, vessels, and nerves (O’Brien et al., 2014; Shi and Vanhoutte, 2017; Rawshani et al., 2018; Parravano et al., 2021). However, one of the more all-encompassing predicaments deemed deleterious for type 1 and type 2 diabetes is the loss of muscle mass, which may lead to mortality or contribute to disability in some cases (reviewed by Bonaldo and Sandri, 2013). STZ-induced diabetes for 8 weeks in mice showed significant atrophy in the gastrocnemius muscle (Liu et al., 2022). In addition, tibialis anterior muscle mass and myofiber cross-sectional area were significantly decreased in STZ-induced diabetic mice with muscle-specific triple knockout of FoxO1/3/4 (O’Neill et al., 2019). Therefore, it is apparent that loss of muscle mass is an observable feature in diabetic patients (O’Neill et al., 2019; Liu et al., 2022). Researchers in this field discovered that a spike in glucose levels and vascular complications cause the loss of muscle mass (Dziubek et al., 2015; Hirata et al., 2019). Muscle maintenance processes result from a delicate balancing act between protein synthesis and degradation at the cellular level (Alber and Suter, 2019). Appell (1990) reviewed muscle strength loss, changes in EMG, muscle weight, muscle fiber size, susceptibility of a specific type of muscle fibers for atrophy, connective tissue contents, oxidative enzyme content, protein metabolism, autophagy and mitochondrial function after immobilization.

10 days of immobilization in humans resulted in significant atrophy in the vastus lateralis muscle, and daily heat therapy decreased such atrophy (Hafen, 2019). Chronic diabetes has been shown to result in muscle atrophy (O’Neill et al., 2019; Liu et al., 2022). Knee joint immobilization for 5 days showed significant atrophy in the quadriceps femoris muscle in human volunteers, and it was counteracted by neuromuscular electrical stimulation (Dirks et al., 2014). Cohen et al. reviewed different types of muscle atrophy and new understandings and therapies that can be used to fight muscle wasting (Cohen et al., 2015).

Under heat stress situations, heat stress protein70 (HSP70) expressed in the muscles stabilizes the structure and function of the skeletal muscle, maintains muscle mass and ultimately attenuates atrophy (Gjovaag et al., 2006; Selsby et al., 2007). As heat stress activates HSPs (Sebők et al., 2021), heat therapy may be applied to immobilized, paralyzed, and atrophying muscles to prevent atrophy. Heat exposure causes microvascular remodeling in muscle tissue, increases glucose absorption, stimulates the production of heat shock proteins, and suppresses inflammatory, lipogenic, and oxidative stress processes (Sebők et al., 2021). The heat shock proteins and other proteins expressed through various mechanisms facilitate muscle mass maintenance (Gjovaag et al., 2006; Selsby et al., 2007). Heat therapy increases core temperature and stresses the body to prevent muscle atrophy by inhibiting the upregulation of MAFbx transcription (Houston et al., 2015). It also allows more natural muscle mass gain, enhanced stress tolerance, higher insulin sensitivity, lower inflammation, and improved cardiovascular and circulatory function (Patrick and Johnson, 2021).

The use of heat therapy, such as sauna, has been in practice for a long time, for better health in human subjects, where the body is exposed to a temperature of 80°C, for 20 min, 4–7 times weekly. In the sauna, the core body temperature increases ∼39°C, as the skin temperature rises to 40°C, resulting in increased cardiac output and heart rate. Therefore, using sauna has been suggested as an alternative to exercising for those who cannot do so due to a chronic illness or physical restrictions (Patrick and Johnson, 2021). Several other heat therapy modalities, such as warm whirlpool baths, wraps and use of physiotherapy machinery which uses short wave diathermy, are in use (Hafen et al., 2019). Megapulse II machine used for diathermy transmits electromagnetic waves into the area of concern such that an internal increase in kinetic energy results in heating. Another heat therapy modality is wet heat or achieving heat using a water bath where heat stress is attained by immersing the hind limbs in a water bath maintained at a temperature of 42°C (Ohira et al., 2017). As exposure of whole-body to heat stress by keeping the rats in an enclosed heat chamber, reduced the hind limb muscle atrophy induced by hindlimb unweighting (Naito et al., 2000), this method of heat therapy which ensures uniform heat over entire body and precise heat levels appears to be most practical method for experimental studies.

Several proteins play vital roles in the diabetes-induced atrophy of muscles (degeneration markers), such as the MAFbx or atrogin-1 ubiquitin ligase and KLF, CD68. The most crucial proteasome involved in muscle atrophy is thought to be the ubiquitin-proteasome system (Gao et al., 2018). It involves the ubiquitin-activating enzyme E1, ubiquitin-conjugating enzyme E2 and ubiquitin ligase E3 (Gao, 2018). Within the E3 proteins, there are two classes, MuRF1 and MAFbx or atrogin-1, which play an essential role in degrading muscle tissue (Gao et al., 2018); FOXO transcription factors regulate this protein (O’Neill et al., 2019). The mechanism by which MAFbx functions is through the degradation of both MyoD, a vital transcription factor for muscle synthesis, and eIF3f, an activator of the protein synthesis cascade (Sanchez et al., 2013). An abundance of transcription factor KLF15 was reported in the skeletal muscle of diabetic mice, and its knockout ceases skeletal muscle atrophy (Hirata et al., 2019). The quantity of infiltrating inflammatory cells, such as CD68, is a reliable indicator of a damaged muscle. High CD68 positive counts are associated with severely inflamed or damaged muscle cells (Howard et al., 2021).

Several other proteins collectively work to improve protein synthesis and suppress atrophic processes (regeneration markers), such as Akt, mTOR, and HSP70. Akt is a serine/threonine kinase that activates mTOR. In DM subjects, significantly reduced activity of Akt kinase was reported compared to healthy controls (Perry et al., 2016). Diminished activity of Akt during DM, which no longer can activate mTOR, results in a considerable reduction of protein synthesis in skeletal muscles. mTOR, which gets activated by Akt *via* insulin, is the primary regulator of protein synthesis in skeletal muscle (Perry et al., 2016). HSP70 is a heat shock protein that is activated during heat and/or stress bouts. HSP70 plays its role in protein synthesis. It receives an unfolded or newly created amino acid chain, aids in its folding into the correct functional form and then releases it (Tupling et al., 2008).

Even though heat therapy proved to have several health benefits, its role in preventing muscle atrophy and proteins involved in muscle degeneration/regeneration is not studied thoroughly in diabetic conditions. We hypothesize that heat therapy, which activates HSPs, and other proteins, may be beneficial in preventing muscular atrophy in diabetes. It will downregulate muscle degeneration markers and upregulate muscle regeneration markers in atrophying muscles, thereby preventing muscular atrophy in diabetic individuals. The present study is aimed to determine the effects of heat therapy in attenuating muscle atrophy and functional deficits and analyze the changes in the levels of expression of CD68, KLF, MAFBx (muscle degeneration markers), Akt, mTOR and HSP70 (muscle regeneration marker) in the STZ-induced diabetic rats. Heat therapy is known to be beneficial in maintaining muscle mass in conditions such as immobilization (Hafen, 2019), paralysis (Chen, and Shaw, 2006), chronic diseases (O’Neill et al., 2019; Liu et al., 2022) and diabetes. We used an STZ-induced diabetic model in this study as an increase in glucose levels and vascular complications following diabetes cause the loss of muscle mass or muscular atrophy (Dziubek et al., 2015; Hirata et al., 2019). Preventing muscle atrophy and maintaining muscle mass is a clinical requirement to provide better health status for diabetic patients. Hence we selected diabetes induced muscle atrophy model for the present study.

## 2 Materials and methods

### 2.1 Animals

Sprague Dawley male rats (3 months of age, ∼300–400 g; N = 96, 48 for short-term and 48 for long-term experiments) were obtained from the Animal Resources Center, Health Sciences Center, Kuwait University. They were housed in plastic cages (Two rats/cage both in control and experimental groups) with free access to food and water *ad libitum*. Rats were fed with rodent maintenance feed with low phytoestrogen (Cat# V1514-300) from ssniff Spezial diäten GmbH (Ferdinand-Gabriel-Weg 16 D-59494 Soest, Germany). The bedding in all cages was hardwood chips that would provide animals with a high absorbance rate and a comfortable environment. All rats were kept under a 12 h light/dark cycle (light from 7 a.m. to 7 p.m.), and the temperature was maintained at 22 ± 2°C to provide them with an ambient environment. All animal experiments were done strictly following the guidelines set by Kuwait University after obtaining animal ethical approval from the Animal Ethics Committee, Health Sciences Center, Kuwait University, Kuwait.

### 2.2 Experimental design

The study was divided into short- and long-term experiments. In both experiments, rats were randomly divided into four groups: i) Control (C), ii) Heat therapy (H), iii) Diabetic (D), and iv) Diabetic + Heat therapy (D + H). For each group, we used 24 rats (12 for short-term and 12 for long-term experiment). Throughout the experiment, rats in the control group remained undisturbed in their home cages. Rats in heat therapy groups (groups ii and iv) were subjected to 30-min daily heat therapy by keeping them in an incubator at 42°C for 3 weeks (short-term), or 6 weeks (long-term). Diabetes was induced in rats in the diabetic groups (groups iii and iv) by injecting a single dose of STZ (60 mg/kg) intraperitoneally. The rats in the control and diabetic groups were exposed to sham heat therapy. Rats in the diabetic + heat therapy group were subjected to heat therapy (exposure to 42°C, 30 min daily for three or 6-weeks). In the present study, we used “short-term” and “long-term” for 3-weeks and 6-weeks study relative to each other even though there is only 3 weeks difference between short-term and long-term interventions. Three-weeks and 6-weeks intervention durations were chosen based on the literature and taking into account the health condition of diabetic rats as diabetes progresses. Previous studies used heat therapy intervention for various conditions for 3–8 weeks (O'Neill et al., 2019; Liu et al., 2022). As it was observed in earlier studies that rats lose body weight and become too weak and die due to prolonged (8–10 weeks) untreated diabetic condition and unadvised to go for more than 6 weeks’ intervention for the above reasons (Renno et al., 2008; Afsari et al., 2008; Dalaklioglu et al., 2014), we limited intervention to the maximum of 6 weeks duration. Rats in all groups were subjected to Kondziela’s inverted wire mesh screen test, rotarod and extensor postural thrust (EPT) neuromuscular functional performance tests. After functional tests, all rats were sacrificed, and quadriceps femoris muscle tissue was collected for morphological and biochemical studies. Muscle wet weight was noted. The morphological study included the histopathological analysis of muscle structure by staining the sections with hematoxylin and eosin, measurement of the cross-sectional area of the muscle fibers, transmission electron microscopy, immunofluorescence staining for expression of CD68, KLF, MAFBx, AKT, mTOR, and HSP70 and fluorescence intensity quantification. By western blot analysis, the biochemical study estimated CD68, KLF, MAFBX, AKT, mTOR, and HSP70 levels in muscle tissue.

### 2.3 Induction of diabetes

Diabetes was induced in diabetic (D) and diabetic + heat therapy (D + H) groups by injecting a single dose of STZ intraperitoneally (60 mg/kg, i.p., Sigma-Aldrich, St. Louis, Missouri, United States) as described earlier (Renno et al., 2008). Briefly, STZ was freshly prepared in ice-cold citrate buffer (pH 4.5). Rats were fasted overnight before injecting STZ for quick absorption. Rats were allowed free access to food and water after STZ injection. To confirm diabetes, blood glucose was checked 48 h after STZ injection using a glucometer (Bayer Corporation, Elkhart, Indiana, United States). The rats with a blood glucose level of more than 200 mg/dl blood were considered diabetic and included in the study (Afsari et al., 2008; Renno et al., 2008).

### 2.4 Heat therapy

Rats in heat therapy groups (H and D + H groups) were placed in a heat controlled chamber maintained at 42°C with a relative humidity of 15.0 ± 2.0% for 30 min daily for 3 weeks (short-term experiment) or 6 weeks (long-term experiment), (Beever et al., 2009; Sobajima et al., 2011; Tamura et al., 2015). Drinking water was available for the rats throughout the heat therapy period. Rectal temperature was recorded in all rats 10 minutes after the commencement of heat exposure (to ensure core temperature was raised effectively) and at the end of heat exposure (to ensure it is maintained at the increased level and not significantly beyond 40°C) on the first 2 days of each week with a calibrated thermistor probe (Shibaura Electronics, Tokyo, Japan) inserted 6–7 cm past the anal sphincter into the rectum to know the core body temperature. The Mean core body temperature measured was 40.84 ± 0.04°C at the beginning and the end of the heat exposure period. Rats groups that are not subjected to heat therapy (Control and Diabetic) were also handled in a similar way as Heat therapy and Diabetic + Heat therapy groups, except for exposing them to heat therapy. They were moved to the same facility where heat therapy was given, and other neuromuscular functional tests were done, and they were also maintained in the same lighting and temperature conditions. They were handled daily by the same experimenter and placed in the heating chamber at room temperature for 30 min. Rectal temperature was measured 10 min after the commencement of the session and at the end of the session. After heat exposure or sham exposure, rats were returned to their home cage in the temperature-controlled (22 ± 2°C) animal house room and provided rat chow and water *ad libitum*.

### 2.5 Motor functional tests

Rats in all groups were subjected to Kondziela’s Inverted wire mesh screen, Rotarod and Extensor Postural Thrust (EPT) neuromuscular behavioral tests.

#### 2.5.1 Kondziela’s inverted screen test

Limbs muscle strength was tested by Kondziela’s inverted screen test (Deacon, 2013). The test apparatus consisted of a square wire mesh of 45 cm × 45 cm dimension, made from stainless steel wire of 1.0 mm diameter. The dimension of each window in the mesh was 12.0 × 12.0 mm. Wire mesh was attached to a wooden frame 4.0 cm high (to prevent the rat from escaping). The rat was placed in the center of the wire mesh, and the mesh was inverted such that the rat was hanging to the mesh with its paw. The mesh was held steadily 40.0 cm above a padded surface. The time the rat took to fall from the mesh (latency of falling) was noted for each rat with a stopwatch. The test ends when the rat falls on its own or terminated by the experimenter at the end of 60-s, giving latency of falling as 60 s.

#### 2.5.2 Rotarod test

The rotarod test, a widely used test to evaluate the neuromuscular motor coordination of rodents, was used to test the motor coordination of rats in different groups. In this test, the rat will be placed on a horizontal rod that rotates around its long axis with an increasing speed. Latency of falling (Time in seconds from starting acceleration to rat falling from the rod) will be recorded for each rat (Monville et al., 2006). The Rotarod test was done on the digital rotarod apparatus (UB-47750 Rat Rota-Rod NG, Ugo Basile SRL, Varese, Italy) as described earlier (Renno et al., 2008). The Rotarod was set at the acceleration mode (initial speed-5rpm/min; maximum speed-45 rpm/min). The rat was placed on the apparatus’s rotating rod and falling latency in each trial was recorded. Three trials were given for each rat, and the mean falling latency was calculated.

#### 2.5.3 Extensor postural thrust (EPT) test

Extensor postural thrust test was used to test the muscle strength (Thalhammer et al., 1995). The test was performed as described earlier (Rodrigues et al., 2020; Renno et al., 2021). Briefly, the rat was held upright with both its hind limbs extended such that the distal metatarsus and toes supported the body weight on a weighing balance, and the amount of thrust exerted by the hind limbs on the weighing balance was recorded in grams. Each rat was given three trials and mean value was used to calculate the motor strength of the muscles.

### 2.6 Muscle tissue sample collection for morphological studies and western blot analysis

At the end of the neuromuscular functional tests, rats were anesthetized with CO_2_ asphyxiation. Quadriceps femoris muscle was collected after perfusing with 50 ml of saline followed by 150 ml of freshly prepared 4% paraformaldehyde for morphological studies. Tissues were post-fixed in the same fixative for 48 h before processing for paraffin section cutting. For western blot analysis, quadriceps femoris muscle tissue was collected after perfusing with 50 ml of cold saline and stored at −80°C until further use.

#### 2.6.1 Tissue processing for histological analysis

The muscle tissues were cut into 5 mm(L) x 5 mm (Breadth) x 8 mm (Height) blocks and dehydrated in ascending grades of ethyl alcohol. Then, tissues were cleared in xylene, paraffin infiltration was done at 65°C, and paraffin blocks were made by orienting the tissue for cross-section. Blocks were cut at 5 µm thickness and mounted on Poly-L-lysine coated glass slides. Sections were stained with hematoxylin and eosin stains. Briefly, sections were deparaffinized in xylene, rehydrated in descending grades of ethyl alcohol, and stained with hematoxylin stain. Sections were washed with running tap water to make the nuclei deep blue. The sections were stained with eosin, dehydrated, cleared, and mounted with DPX (Fluka Chemika, Switzerland) and visualized using an Olympus microscope (DP-72; Olympus, Tokyo, Japan).

#### 2.6.2 Determination of muscle fiber cross-sectional area

Muscle fiber cross-sectional area (CSA) was measured on photographs of H&E stained sections. Photomicrographs of muscle cross-sections were captured at 40X magnification as digital images using an Olympus microscope, with digital camera (DP-72) and software. Randomly selected six photographs from the mid-belly region of the quadriceps femoris muscle were used for cross-sectional area measurement. Using calibrated Image-J software, the cross-sectional area of a total of 50 muscle fibers/muscle was measured in each tissue sample, and the mean CSA was calculated.

#### 2.6.3 Tissue processing for electron microscopy

For electron microscopy, muscle samples were fixed in 2.5% glutaraldehyde in 0.1 M sodium cacodylate buffer (pH 7.3) for 24 h and then washed four times in the sodium cacodylate buffer. Tissue was then post-fixed with 1% osmium tetroxide for 60 min at room temperature. The muscle tissue samples were washed twice in distilled water, dehydrated *via* a graded series of propylene oxide, infiltrated with a graded combination of propylene oxide and Epon and finally embedded in 100% Epon. The ultra-thin sections were cut (using a Leica Ultracut UCT ultramicrotome; Rowako AB, Vendelsö, Sweden). The contrast was enhanced by staining with 0.1% uranyl acetate and lead citrate (FEI Company, Eindhoven, Netherlands). Philips EM 208 electron microscope attached with Megaview III FW camera was used to take electron photomicrographs.

#### 2.6.4 Immunohistochemistry

The immunohistochemistry technique was used to assess the expression of the muscle proteins. Cross-sections of the muscle were immunostained for markers such as CD68, KLF, MAFBx, AKT, mTOR, and HSP70. All these markers were visualized with fluorescent immunostaining technique with respective primary antibodies (mouse anti-CD68(sc-20060), anti-KLF (sc-271675), anti-MAFBx (sc-166806), anti-AKT (sc-81434), anti-mTOR(sc-517464), anti-HSP70(sc-32239), (All primary antibodies are from Santa Cruz Biotechnology, Inc., United States). As previously described, rat muscle tissues were processed for immunohistochemistry (Al-Adwani et al., 2019). The muscle tissues were collected and cut into 3 mm thick blocks. Then 5 μm thick sections (10 sections/rat) were cut, mounted on Poly-L-lysine coated glass slides, kept overnight for air drying and the sections were immunostained. Briefly, sections were deparaffinized, rehydrated and antigen retrieval was done using 0.1 M citrate buffer (pH = 6.5) at 60°C for 15 min. Sections were then blocked for 30 min with 5% normal horse serum (Cat. No. H0146; Sigma-Aldrich, Saint Louis, Missouri, United States) containing 0.01% Triton X-100 in PBS. Then the sections were incubated in the different primary antibodies (1:500) at 4°C for overnight. The sections were then washed and incubated in secondary antibody (rabbit anti-mouse) conjugated with fluorochrome at room temperature for 1 h (in dark). Finally, the sections were mounted and visualized in a confocal microscope.

### 2.7 Western blot analysis for muscle proteins (CD68, KLF, Atrogin/MAFbx, Akt, mTOR, and HSP70)

Western blot analysis for CD68, KLF, Atrogin/MAFbx, Akt, mTOR, and HSP70 proteins were performed as described earlier (Renno et al., 2021). Samples were homogenized with buffer containing 10 mM MgCl2, 10 mM KH2PO4, 1 mM EDTA, 5 mM EGTA, 1% Igepal, 50 mM βGPO4, 1 mM PMSF, 1 mM Na3VO4, 1 μg/ml leupeptin, 2 μg/ml antipain, 10 μg/ml benzamidine, 1 μg/ml aprotinin, 1 μg/ml chymostatin, and 1 μg/ml pepstatin. After homogenization, samples were centrifuged for 10 min at 14,000 rpm at 4°C, and the supernatant was collected. The protein concentration of the supernatants was determined by the Bradford method using the Bio-Rad (Hercules, CA) protein assay reagent. 75 μg of the protein was loaded onto ready-made Bio-Rad gradient (4–20%) polyacrylamide gels. After electrophoretic separation, proteins were transferred to nitrocellulose membranes. Subsequently, the membranes were stained in Ponceau-S solution (Sigma, St. Louis, MO) for 5 min for visualization of the protein bands and assurance of equal loading of samples in all the lanes. Membranes were blocked in Odessey blocking buffer (Licor, Lincoln, NE) for 2 h at room temperature and incubated in primary antibody overnight in Odessey blocking buffer with 0.1% tween-20. The primary antibody for all proteins under study are mouse monoclonal IgG (Santa Cruz Biotech) and used at 1:100 dilutions. After three washes, membranes were incubated in rabbit anti-mouse IgG (Santa Cruz Biotech) at a concentration of 1:2,500 for 1 h at room temperature. The membranes were later incubated with Western blotting luminol reagent (Santa Cruz Biotech) in the dark room for 5 min, and the membrane was exposed to x-ray film. The films were subsequently developed and fixed. Comparisons were made against a protein marker ladder in order to determine if the protein being visualized is in fact the target protein based on molecular weight. Densitometry was performed to quantify the bands using ImageJ software.

### 2.8 Statistical analysis

To test statistical differences between the groups, one-way or two-way ANOVA was used wherever applicable; if significant F-ratios was observed, Bonferroni’s multiple comparisons test was used to test individual means. Statistical significance was assumed at *p* < 0.05. SPSS statistical package version 21 (IBM, United States) was used for statistical analysis.

## 3 Results

### 3.1 Body temperature

Core body temperature measured 10 min after the beginning of the session and at the end of the session showed a significant increase in heat therapy groups compared to control groups at all weeks (W1-6) both in 3 weeks and 6 weeks experiments (*p* < 0.001, Tables 1, 2). Similarly, core body temperature measured 10 min after the beginning of the session and at the end of the session in Diabetic + Heat groups increased significantly compared to diabetic groups at all weeks (W1-6) both in 3 weeks and 6 weeks experiments (*p* < 0.001, Tables 1, 2). Furthermore, we did not find any fluctuation in the core body temperature due to acclimatization.

**TABLE 1:** Core body temperature (°C) (Mean ± SEM) measured 10 min after beginning of the session.

Core body temperature (°C) (Mean ± SEM)				
(Recorded 10 min after beginning of the session)				
Week	Control	Heat	Diabetic	Diabetic + Heat
W-1(n=24/group)	37.29 ± 0.24	37.54 ± 0.18	37.38 ± 0.16	37.29 ± 0.18
Wl(n=24/group)	37.67 ± 0.22	40.63 ± 0.16^***^	37.00 ± 0.18	40.54 ± 0.24^###^
W2(n=24/group)	37.13 ± 0.20	40.96 ± 0.14^***^	36.92 ± 0.18	40.54 ± 0.21^###^
W3(n=24/group)	37.13 ± 0.22	40.96 ± 0.12^***^	36.92 ± 0.20	40.54 ± 0.16^###^
W4(n=12/group)	37.17 ± 0.21	40.83 ± 0.21^***^	36.42 ± 0.29	40.75 ± 0.18^###^
W5(n=12/group)	37.25 ± 0.22	40.83 ± 0.17^***^	36.42 ± 0.23	40.92 ± 0.19^###^
W6(n=12/group)	36.58 ± 0.31	40.83 ± 0.21^***^	36.92 ± 0.23	40.42 ± 0.15^###^
Mean (W1-W6)	37.155 ± 0.14	40.84 ± 0.04	36.76 ± 0.11	40.61 ± 0.07

Note significantly increased core temperature in heat therapy (Heat) group compared to control group (***Control Vs. Heat, *p* < 0.001) and in Diabetic + Heat compared to diabetic group; (### Diabetic Vs. Diabetic + Heat, *p* < 0.001); in W1-W6.

**TABLE 2:** Core body temperature (°C) (Mean ± SEM) measured at the end of the session.

Core body temperature (°C) (Mean ± SEM)(Recorded at the end of the session)				
Week	control	Heat	Diabetic	Diabetc+Heat
W-1(n=24/group)	37.17±0.25	37.25±0.23	37.38±0.20	37.67±0.22
W1(n=24/group)	37.29±0.18	40.54±0.24***	36.92±0.22	40.63±0.16^###^
W2(n=24/group)	37.13±0.20	40.96±0.14***	37.00±0.18	40.54±0.21^###^
W3(n=24/group)	36.92±0.22	40.71±0.16***	37.13±0.20	40.79±0.12^###^
W4(n=12/group)	37.33±0.22	40.83±0.17***	36.42±0.29	40.83±0.21^###^
W5(n=12/group)	37.17±0.21	40.92±0.19***	36.42±0.23	40.83±0.17^###^
W6(n=12/group)	36.92±0.23	40.50±0.15***	36.58±0.31	40.83±0.21^###^
Mean(W1-W6)	37.12±0.07	40.74±0.07	36.74±0.12	40.70±0.05

Note significantly increased core temperature in heat therapy (Heat) group compared to control group (***Control Vs. Heat, *p* < 0.001) and in Diabetic + Heat compared to diabetic group; (### Diabetic Vs. Diabetic + Heat, *p* < 0.001); in W1-W6.

### 3.2 Blood glucose level

**FIGURE 1:**
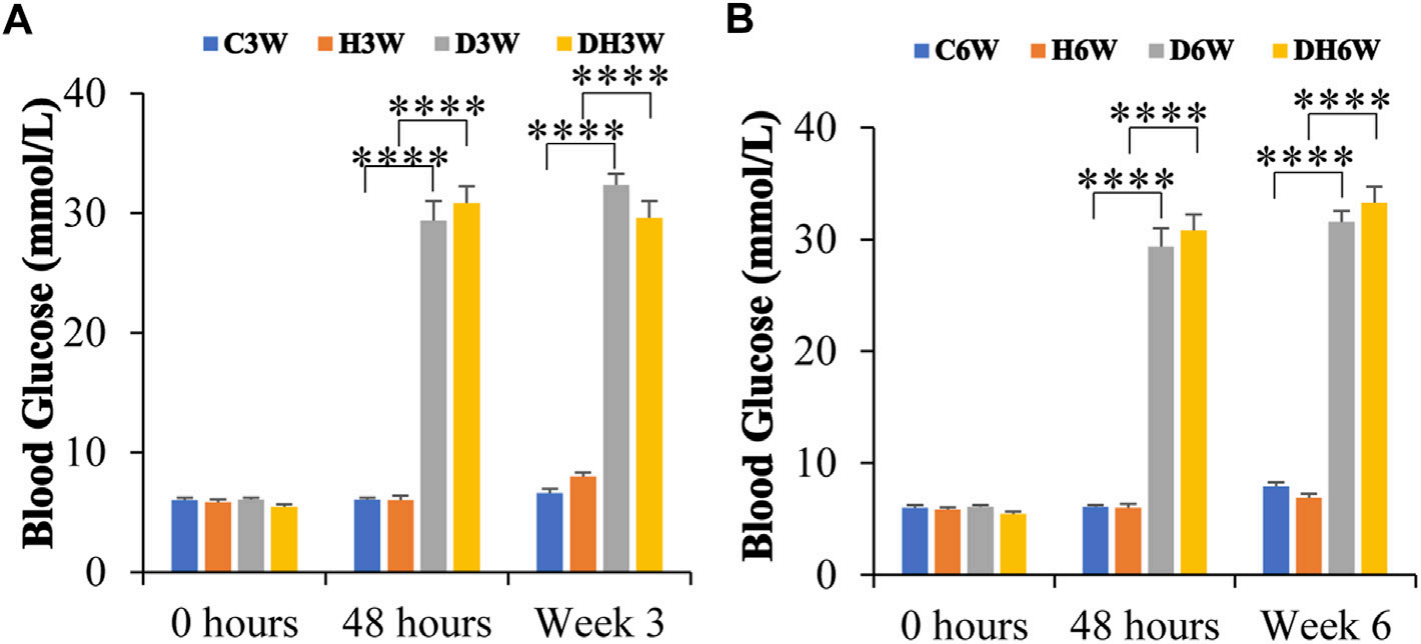
Blood glucose levels of rats in different groups in short-term **(A)** and long-term **(B)** experiments. Note that there were no significant differences in blood glucose levels between groups before STZ injection (0 h) in both short and long-term experiments. However, the diabetic and diabetic + heat therapy group of rats (D3W, DH3W, D6W, and DH6W) showed a significant increase in blood glucose compared to respective control groups at all-time points of measurements (48 h, 3 and 6 weeks) after STZ injection (****, *p* < 0.0001, One-way ANOVA, Bonferroni multiple comparison tests, *n* = 6 in all groups) and there was no difference in blood glucose level between short- and long-term experiments.

At the beginning of the experiment, before STZ injection (0 h), blood glucose was within the normal range (5.9–6.5 mmol/L) in all groups of experimental animals and statistical analysis did not show any significant difference between any groups (*p* > 0.05, Figures 1A,B). The blood glucose level in C3W, C6W, H3W, and H6W remained in the normal range throughout the experiment. Forty-8 hours after STZ injection, D3W, D6W, DH3W and DH6W groups showed a significant increase in blood glucose levels (29 mmol/L - 32 mmol/L) compared to respective control groups indicating a severe diabetic condition (*p* < 0.0001, One-way ANOVA, Bonferroni multiple comparison tests, n = 6 in all groups). The blood glucose level at the end of the third week (in D3W and DH3W) and even at end of 6^th^ week (in D6W and DH6W) remained significantly high compared to respective control groups (*p* < 0.0001, 29 mmol/L- >33.3 mmol/L, Figures 1A,B).

### 3.3 Body weight and quadriceps femoris muscle weight

**FIGURE 2:**
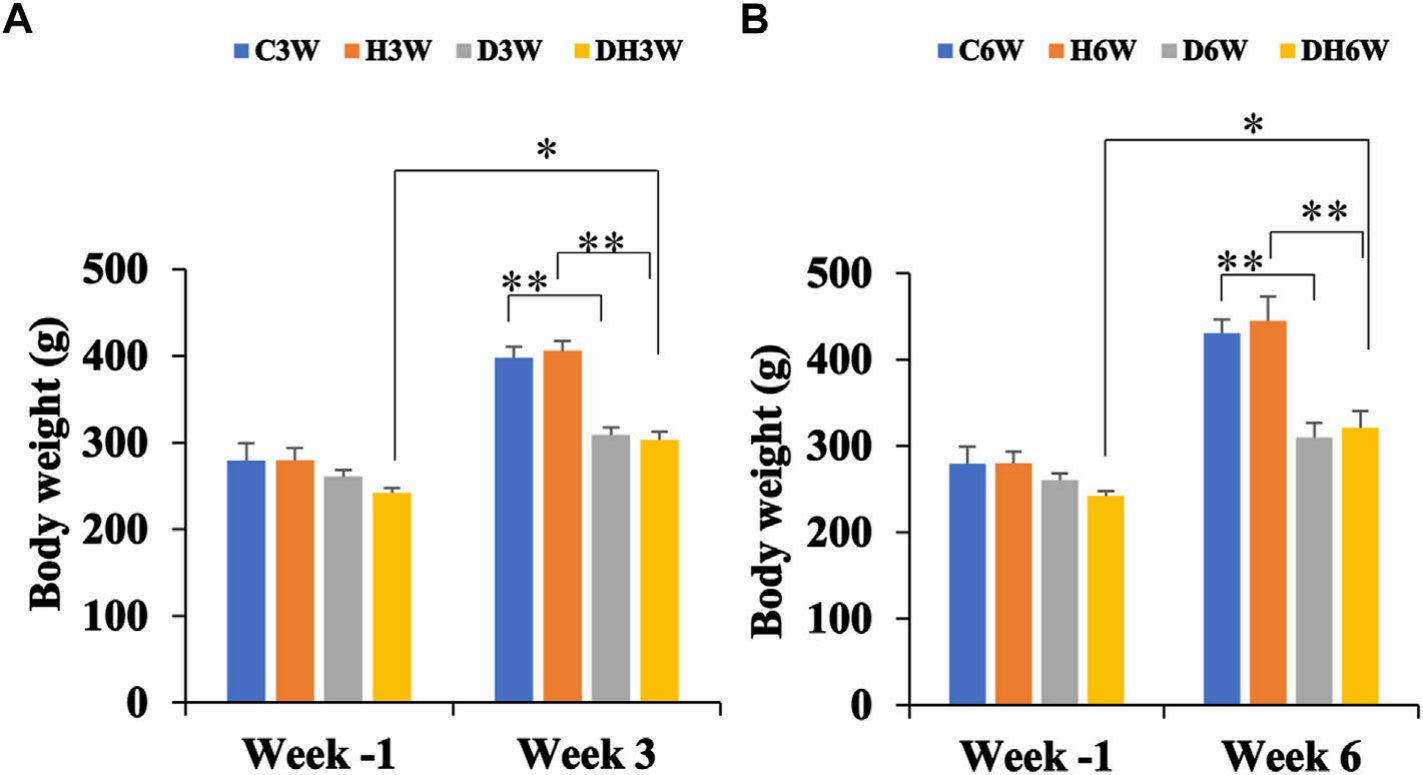
Body weight of rats in different groups in short-term **(A)** and long-term **(B)** experiments. Note there were no significant differences in body weight between groups at week-1 in both short and long term experiments. However, there was a significant decrease in the body weight of rats in diabetic and diabetic + heat therapy groups (D3W, DH3W, D6W, and DH6W) compared to respective control groups both in short and long-term experiments (**p < 0.01, One-way ANOVA, Bonferroni multiple comparison tests, *n* = 6 in all groups). There was no significant difference in body weight loss when compared between diabetic and diabetic + heat therapy groups in both short and long-term experiments. Body weight gain was significantly high in DH3W and DH6W compared to their Week-1 body weight (*, *p* < 0.05)

Body weights in both short-and long-term experiments were monitored throughout the experiment. Before the commencement of the experiment (week -1), there was no significant difference in body weight between the different groups (Figure 2). Body weight of diabetic rats in short-term (D3W and DH3W) and long-term (D6W and DH6W) was found to be significantly less compared to respective control groups (C3W vs. D3W, *p* < 0.01; H3W vs. DH3W, *p* < 0.01; C6W vs. D6W, *p* < 0.01; H6W vs. DH6W, *p* < 0.01, One-way ANOVA, Bonferroni multiple comparison tests, *n* = 6 in all groups). Interestingly, in both short-term (D3W) and long-term (D6W), diabetic rats gained 18% over their body weight prior to STZ injection, whereas diabetic rats subjected to heat therapy, DH3W and DH6, gained 25% and 32% respectively over their body weight prior to STZ injection suggesting beneficial effect of heat therapy in body weight gain in diabetic rats (*p* < 0.05, Figures 2A,B).

The wet weight of quadriceps femoris muscle was significantly decreased in diabetic groups (D3W and D6W) compared to respective control groups (3.29 ± 0.01 g in C3W Vs 2.28 ± 0.03 g in D3W, *p* < 0.001; 3.52 ± 0.01 in C6W Vs 2.43 ± 0.01 in D6W, *p* < 0.001, Table 3). Quadriceps femoris muscle weight was significantly increased in diabetic rats exposed to heat therapy (DH3W and DH6W) compared to respective diabetic groups (2.28 ± 0.03 g in D3W Vs 2.51 ± 0.01 in DH3W, *p* < 0.001; 2.43 ± 0.01 in D6W Vs, 2.66 ± 0.01 in DH6W, *p* < 0.001, Table 3). These results suggest significant muscle atrophy in diabetic rats and heat therapy could counteract the muscle atrophy.

**TABLE 3:** Wet weight of the quadriceps femoris muscle (g) (Mean ± SEM) measured at the time of tissue collection for morphological and biochemical studies. Note significantly decreased wet weight of the quadriceps femoris muscle in diabetic groups (D3W and D6W) compared to respective control groups (***, *p* < 0.001).

Wet weight of Quadriceps femoris muscle (g) (Mean ± SEM)			
Groups	Week3	Groups	Week6
C3W(n=12)	3.29 ± 0.01	C6W(n=12)	3.52 ± 0.01
H3W(n=12)	3.29 ± 0.02	H6W(n=12)	3.51 ± 0.03
D3W(n=12)	2.28 ± 0.03^***^	D6W(n=12)	2.43 ± 0.01^***^
DH3W(n=12)	2.51 ± 0.01^###^	DH6W(n=12)	2.66 ± 0.014^###^

The Quadriceps femoris muscle weight was significantly increased in diabetic rats exposed to heat therapy (DH3W and DH6W) compared to respective diabetic groups (###, *p* < 0.001).

### 3.4 Neuromuscular motor performance

#### 3.4.1 Kondzeila’s inverted screen test

**FIGURE 3:**
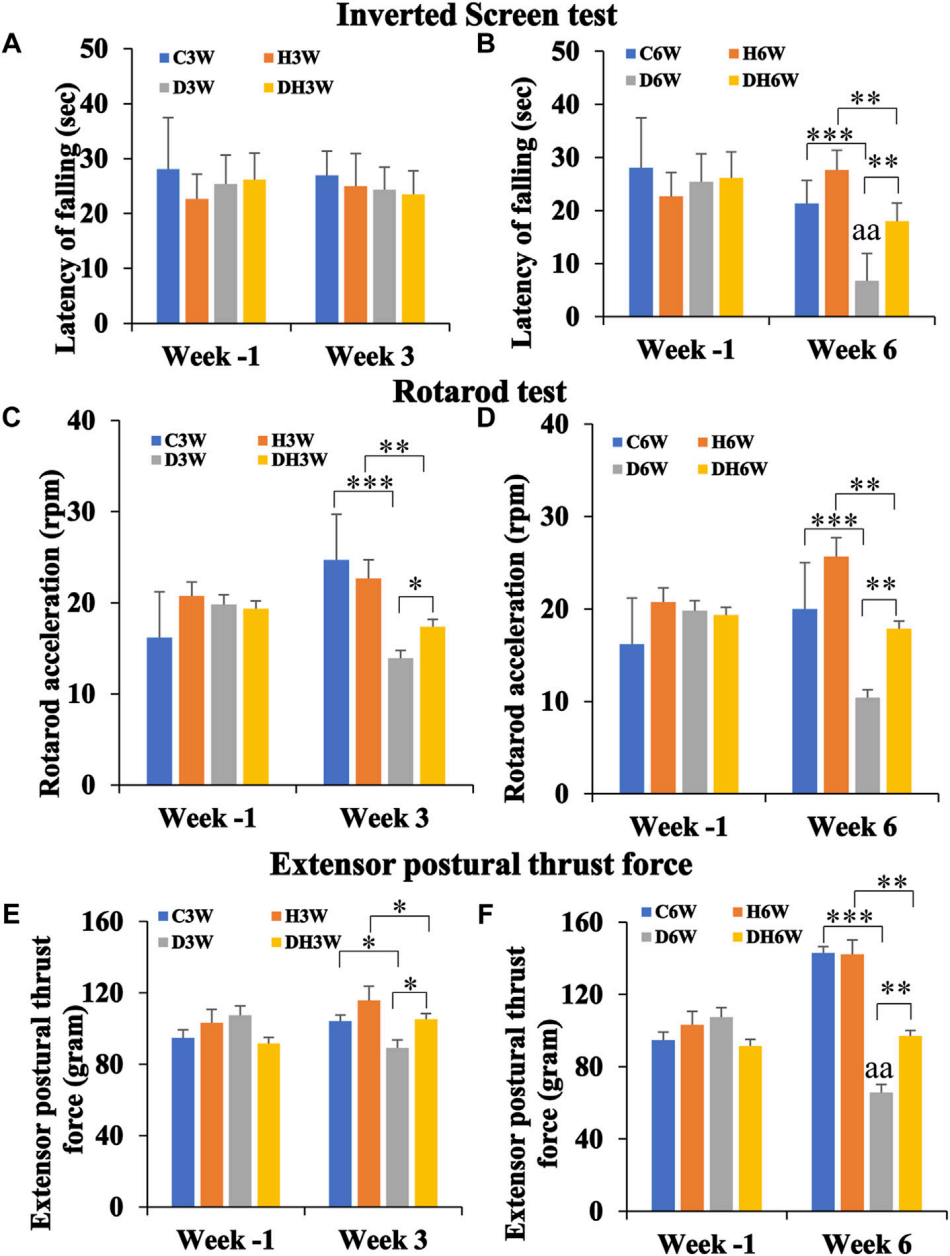
Kondziela’s inverted screen test **(A,B),** Rotarod test **(C,D)** and Extensor postural thrust force test **(E,F)** results. In all the above motor functional tests, there were no significant differences between groups when tested at week-1 [both in the short term **(A,C, and E)** and long-term **(B,D, and F)**]. In both short and long-term experiments, all test parameters (latency of falling of rats in Kondziela’s inverted screen test, rotarod acceleration in rotarod test and extensor postural thrust force) were significantly decreased in diabetic and diabetic + heat therapy groups compared to respective control groups (C3W Vs. D3W, H3W Vs. DH3W; C6W Vs. D6W, H6W Vs. DH6W) except for latency of falling in short term experiment (*p* < 0.05–0.001). Interestingly, in short, and long-term experiments, all test parameters in DH3W and DH6W significantly increased compared to D3W and D6W, respectively(*p* < 0.05–0.01; *, *p* < 0.05; **, *p* < 0.01; ***, *p* < 0.001; D3W vs. D6W-^aa^ *p* < 0.01; One-way ANOVA, Bonferroni multiple comparison tests, n = 6 in all groups).

In this test, rats were hung upside down from a steel wire mesh screen to test their forelimb muscle strength in terms of how long they could hold on, without falling from the mesh. The Latency of falling from mesh collected a week before the start of the experiment (Week -1) did not show any statistical difference between groups in both short- and long-term experiments (Figures 3A,B). In the short-term experiment, C3W, H3W, D3W and DH3W rats held on to the mesh for 27.00 ± 6.56, 25.00 ± 5.94, 24.35 ± 4.11 and 23.5 ± 4.30 s, respectively, with no significant difference between groups. However, in the long-term experiment D6W and DH6W held on to the mesh for a significantly shorter time compared to the respective control groups (21.33 ± 4.91 s in C6W vs., 6.8 ± 1.18 s in D6W, *p* < 0.001; 27.66 ± 9.52 s in H6W vs. 18.00 ± 3.25 s in DH6W, *p* < 0.01, One-way ANOVA, Bonferroni multiple comparison tests, n = 6 in all groups, Figure 3B). Interestingly, the diabetic rats subjected to heat therapy (DH6W) held on to the mesh significantly longer time than D6W could hold suggesting increased muscle strength in them (D6W vs. DH6W, *p* < 0.01). Latency of falling from mesh in diabetic rats was significantly decreased from 3W to 6 W (D3W vs. D6W, *p* < 0.01) but not in diabetic rats subjected to heat therapy (DH3W vs. DH6W, *p* > 0.05, Figures 3A,B).

#### 3.4.2 Rotarod test

In the rotarod test, the rats were tested on a rotating rod that steadily increasing its rotation speed (rpm) to test their ability to hold on and keep up with the accelerating rod without falling. Rota rod acceleration at which rats fell from the rotating rod measured a week before the start of the experiment (Week -1) did not show any statistical difference between groups in both short- and long-term experiments (Figures 3C,D). In the short-term experiment, D3W and DH3W fell from the rotating rod of the rotarod apparatus at a significantly slower acceleration compared to respective control groups (24.71 ± 2.5 rpm in C3W vs., 13.9 ± 0.98 rpm in D3W, *p* < 0.001; 22.66 ± 2.02 rpm in H3W vs. 17.35 ± 0.65 rpm in DH3W, *p* < 0.01, One-way ANOVA, Bonferroni multiple comparison tests, n = 6 in all groups, Figure 3C). Interestingly, the diabetic rats subjected to heat therapy (DH3W) remained on the rotating rod at significantly higher rpm than that of the D3W group suggesting increased muscle strength in them (D3W vs. DH3W, *p* < 0.05). Results of the long-term experiment at week 6, followed the same trend as in the short-term experiment. D6W and DH6W fell from the rotating rod of the rotarod apparatus at a significantly slower acceleration compared to respective control groups (20.0 ± 2.8 rpm in C6W vs., 10.4 ± 0.6 rpm in D6W, *p* < 0.001; 25.66 ± 0.88 rpm in H6W vs. 17.87 ± 0.61 rpm in DH6W, *p* < 0.01, One-way ANOVA, Bonferroni multiple comparison tests, n = 6 in all groups, Figure 3D). Interestingly, the diabetic rats subjected to heat therapy (DH6W) remained on the rotating rod at significantly higher rpm than that of the D6W group suggesting increased muscle strength in them (D6W vs. DH6W, *p* < 0.01). Rotarod acceleration at which rats fell in diabetic rats did not differ significantly from 3 W to 6 W both in diabetic (D3W vs. D6W, *p* > 0.05) and diabetic rats subjected to heat therapy (DH3W vs. DH6W, *p* > 0.05, Figures 3C,D).

#### 3.4.3 Extensor postural thrust test (EPT)

In the EPT test, rats were made to stand upright on a weighing balance to get the amount of force (in grams) exerted by the hind limb extensor muscles thrust force on the balance. Extensor postural thrust force exerted by the rats a week before the start of the experiment (Week -1) did not show any statistical difference between groups in both short- and long-term experiments (Figures 3E,F). In the short-term experiment, D3W and DH3W exerted significantly less extensor postural thrust compared to respective control groups (104.16 ± 3.02 g in C3W vs., 89.15 ± 2.43 g in D3W, *p* < 0.05; 115.83 ± 4.86 g in H3W vs. 105.25 ± 2.54 g in DH3W, *p* < 0.05, One-way ANOVA, Bonferroni multiple comparison tests, n = 6 in all groups, Figure 3E). Interestingly, the diabetic rats subjected to heat therapy (DH3W) exerted a significantly higher extensor postural thrust force than that of the D3W group suggesting increased muscle strength in them (D3W vs. DH3W, *p* < 0.05). Results of the long-term experiment at week 6, followed the same trend as in short-term experiment. D6W and DH6W groups of rats exerted significantly less extensor postural thrust force compared to respective control groups (143.0 ± 2.3 g in C6W vs., 65.7 ± 2.92 g in D6W, *p* < 0.001; 142.33 ± 2.66 g in H6W vs. 97.12 ± 3.70 g in DH6W, *p* < 0.01, One-way ANOVA, Bonferroni multiple comparison tests, *n* = 6 in all groups, Figure 3F). Interestingly, the diabetic rats subjected to heat therapy (DH6W) exerted significantly higher extensor postural thrust force than that of the D6W group suggesting increased muscle strength in them (D6W vs. DH6W, *p* < 0.01). Extensor postural thrust force in diabetic rats was significantly decreased from 3W to 6 W (D3W vs. D6W, *p* < 0.01) but not in diabetic rats subjected to heat therapy (DH3W vs. DH6W, *p* > 0.05, Figures 3E,F).

### 3.5 Light and electron microscopic morphology of muscle fibers

**FIGURE 4:**
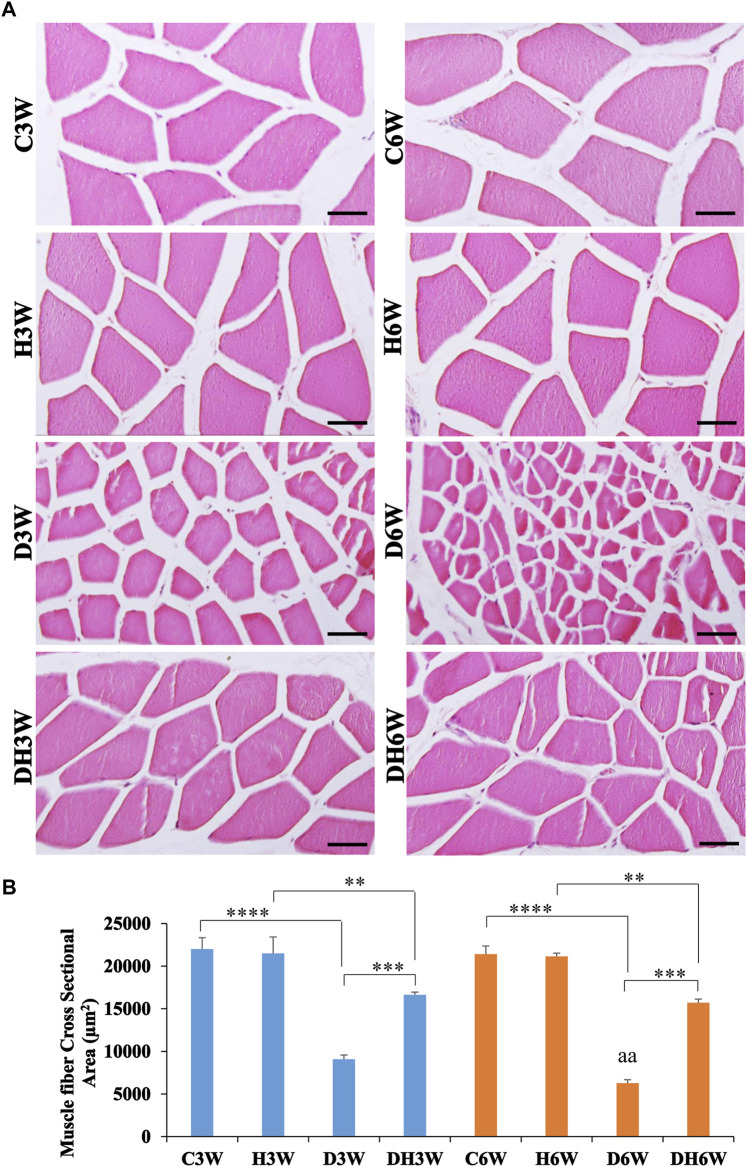
**(A)** Representative photomicrographs of cross sections muscle stained with H&E stain and **(B)** Cross-sectional area of muscle fibers in different groups. Note the atrophied muscle fibers in D3W, which are further atrophied in D6W and such muscle fiber atrophy was reduced in DH3W and DH6W groups. Note the significantly decreased cross-sectional area of muscle fibers in D3W and DH3W compared to C3W and H3W respectively and it was significantly increased in DH3W compared to D3W. A similar trend of the cross-sectional area of muscle fibers was also found in the long-term experiment. Scale bar = 50 µm. (**, *p* < 0.01; ***, *p* < 0.001; ****, *p* < 0.0001, D3W vs. D6W-^aa^ *p* < 0.01; One-way ANOVA, Bonferroni multiple comparison tests, *n* = 6 in all groups).

Cross-sectional area of the quadriceps femoris muscle fibers stained with H&E and viewed under the light microscope showed a remarkable decrease in the size of the muscle fibers in the D3W and D6W groups compared to their respective groups (Figure 4A). In contrast, the DH3W and DH6W groups showed a noticeable increase in muscle fibers size compared to the diabetic controls (D3W and D6W). Analysis of the cross-sectional area of the muscle fibers (CSA) of short-term experiment showed a significant decrease in the cross-sectional area of the muscle fibers in D3W and DH3W compared to C3W and H3W respectively [C3W(22010.67 ± 1318.78 μm^2^) vs. D3W(9089.47 ± 484.45 μm^2^), *p* < 0.0001; H3W 21495 ± 1939.88 μm^2^) vs. DH3W(16637.15 ± 325.01 μm^2^), *p* < 0.01, One-way ANOVA, Bonferroni’s multiple comparison tests, *n* = 6 in all groups, Figure 4B]. Interestingly, the cross-sectional area of muscle fibers of rats subjected to heat therapy was significantly increased compared to diabetic rats (D3W vs. DH3W, *p* < 0.001 Figure 4B). Analysis of the cross-sectional area of the muscle fibers (CSA) of long term experiment also showed a significant decrease in the cross-sectional area of the muscle fibers in D6W and DH6W compared to C6W and H6W respectively [C6W(21421.13 ± 949.23 μm^2^) vs. D6W(6279.56 ± 391.78 μm^2^), *p* < 0.0001; H6W(21154 ± 378.07 μm^2^) vs. DH6W (15722.71 ± 407.68 μm^2^), *p* < 0.01, One-way ANOVA, Bonferroni’s multiple comparison tests, *n* = 6 in all groups, Figure 4B]. Interestingly, the cross-sectional area of muscle fibers of rats subjected to heat therapy was significantly increased compared to diabetic rats (D6W vs. DH6W, *p* < 0.001 Figure 4B). The cross-sectional area of muscle fibers in diabetic rats was significantly decreased from 3 W to 6 W (D3W vs. D6W, *p* < 0.01) but not in diabetic rats subjected to heat therapy (DH3W vs. DH6W, *p* > 0.05, Figure 4B).

**FIGURE 5:**
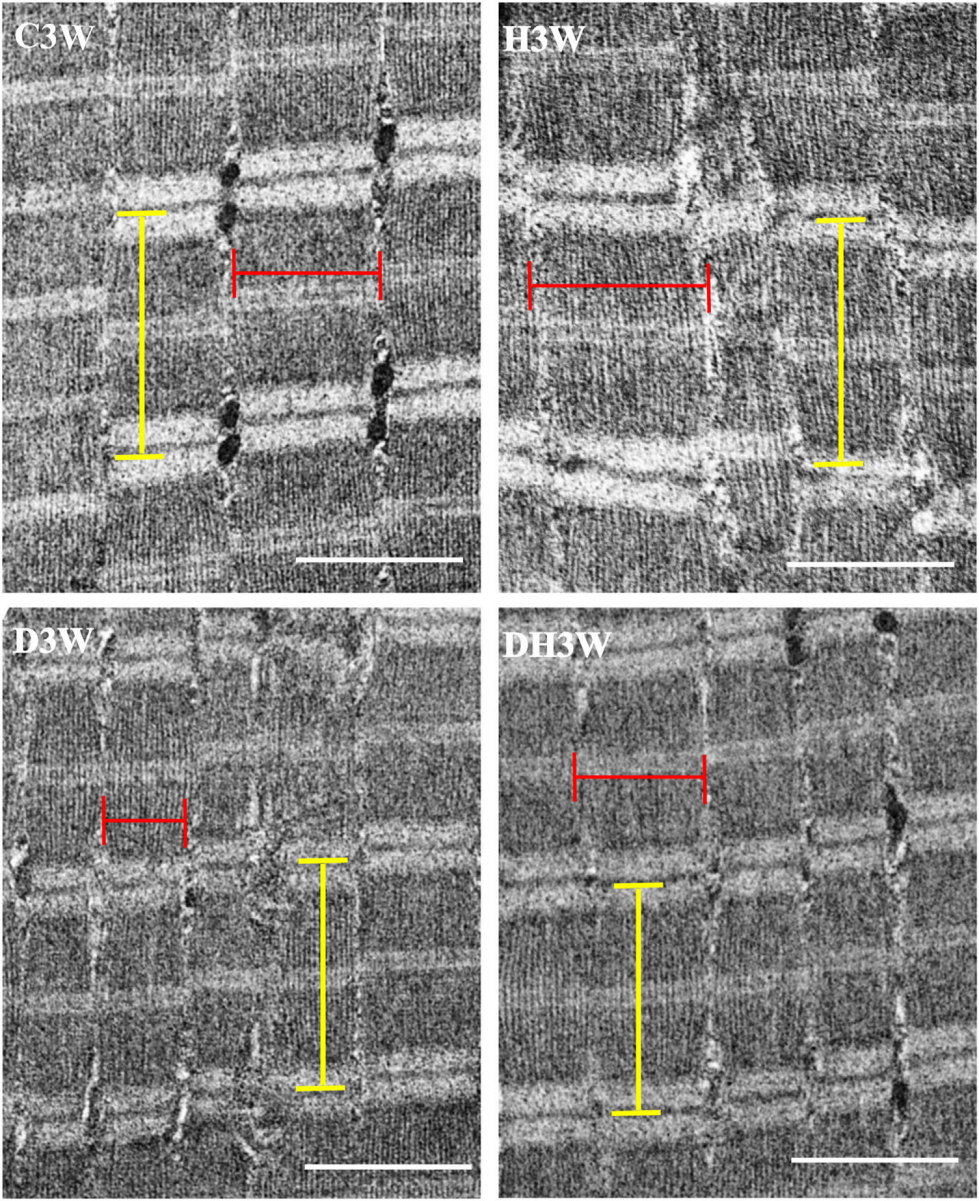
Representative electron photomicrographs of longitudinal sections muscle fibers in different groups in a short term experiment. Note the decreased muscle fibril size (indicated by the red line) in D3W compared to C3W. The muscle fibril size was increased in size in DH3W compared to D3W. However, it is thinner than that in C3W and H3W groups. The dimension of the sarcomere (indicated by the yellow line) was similar when compared between groups. Scale bar = 1000 nm.

**FIGURE 6:**
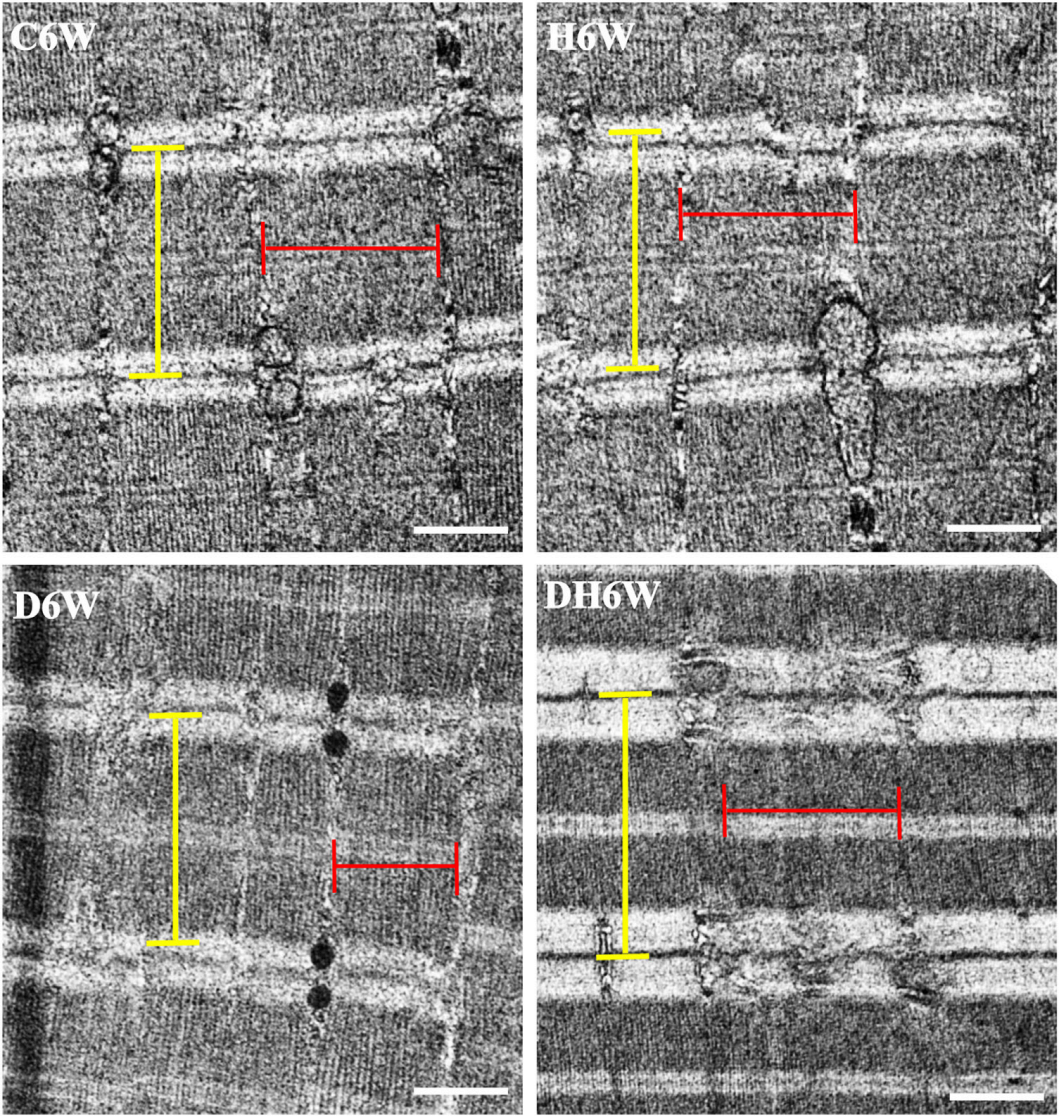
Representative electron photomicrographs of longitudinal sections muscle fibers in different groups in a long-term experiment. Note the decreased muscle fibril size (indicated by red line) in D6W compared to C6W. The muscle fibril size was increased in size in DH6W compared to D6W, and it is comparable to that in C6W and H6W groups. The dimension of the sarcomere (indicated by the yellow line) is decreased in D6W compared to C6W and it is increased in DH6W. Scale bar = 500 nm.

Longitudinal sections of the quadriceps femoris muscle fibers processed for transmission electron microscopy and viewed under a transmission electron microscope showed a noticeable decrease in the breadth of the muscle fibrils in the D3W and D6W groups compared to their C3W and C6W groups (Figures 5, 6). In contrast, the DH3W and DH6W groups showed a noticeable increase in the muscle fibril size compared to H3W and H6W groups. There was no difference in the sarcomere length in different groups of the short-term experiment (Figure 5). However, in D6W, the sarcomere length was decreased in size compared to C6W and it was brought to a size comparable to C6W in rats subjected to heat therapy (DH6W, Figure 6).

### 3.6 Expression of muscle degenerative biomarker proteins (CD68, KLF and MAFbx) in skeletal muscle tissue

#### 3.6.1 CD68

**FIGURE 7:**
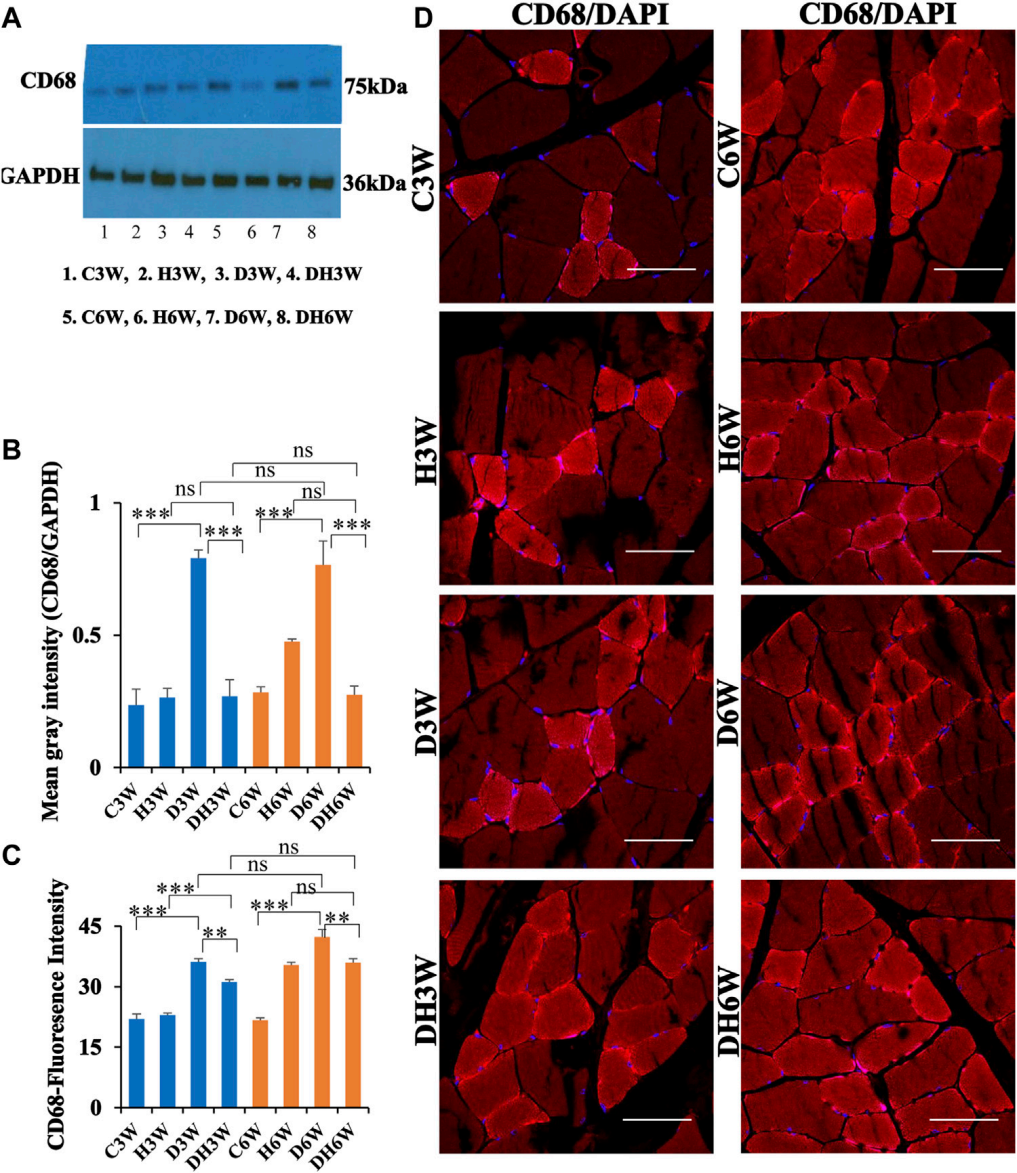
**(A,B)** Immunoblots probed with anti-CD68 antibody **(A)** and the mean gray intensity of immunoblots **(B)**. Note the significantly increased expression of CD68 (mean gray intensity) in D3W and D6W compared to C3W and C6W respectively. CD68 expression was decreased in DH3W and DH6W compared to D3W and D6W respectively. There was no significant difference in CD68 expression from short-term (D3W and DH3W) to long-term (D6W and DH6W). **(C,D)** CD68 immunofluorescence intensity **(C)** and representative cross-section of muscle fibers stained for anti-CD68 immunofluorescence in short-term (left panel) and long-term (right panel) experiments **(D)**. Western blot data of CD68 expression was further supported by fluorescence intensity measured on the anti-CD68 immunostained sections of the muscle fibers in most of the comparisons. Note that many muscle fibers show immunofluorescence in D3W, DH3W, D6W, and DH6W (***, *p* < 0.001, One-way ANOVA, Bonferroni multiple comparison tests, *n* = 6 in all groups). Scale bar = 50 µm.

Western blot analysis for expression of CD68 protein in the skeletal muscle fibers showed a significant increase in diabetic groups both in short-and long term experiments compared to control groups (C3W vs. D3W, *p* < 0.001; C6W vs. D6W, *p* < 0.001; One-way ANOVA, Bonferroni multiple comparison tests, n = 6 in all groups, Figures 7A,B). However, such an increased expression of CD68 was not found in diabetic rats subjected to heat therapy both in short- and long-term experiments compared to respective control groups (H3W vs. DH3W, *p* > 0.05; H6W vs. DH6W, *p* > 0.05). Expression of CD68 was significantly decreased in diabetic rats subjected to heat therapy compared to diabetic rats, that were not subjected to heat therapy both in short- and long-term experiments (D3W vs. DH3W, *p* < 0.001; D6W vs. DH6W, *p* < 0.001). There was no significant difference in the expression of CD68 in short-term diabetic and diabetic and heat therapy groups compared to respective long term groups (D3W vs. D6W, *p* > 0.05; DH3W vs. DH6W, *p* > 0.05, Figures 7A,B). Western blot analysis data of CD68 was supported by immunofluorescence intensity data measured on the cross sections of the muscle fibers stained for anti-CD68 immunofluorescence both in short- and long-term experiments (Figure 7C). The cross sections of the skeletal muscle, stained for anti-CD68 immunofluorescence, showed many positive immunostained muscle fibers in diabetic rats (Figure 7D, 3^rd^ row) and their expression decreased in diabetic rats subjected to heat therapy (Figure 7D, 4^th^ row).

#### 3.6.2 KLF

**FIGURE 8:**
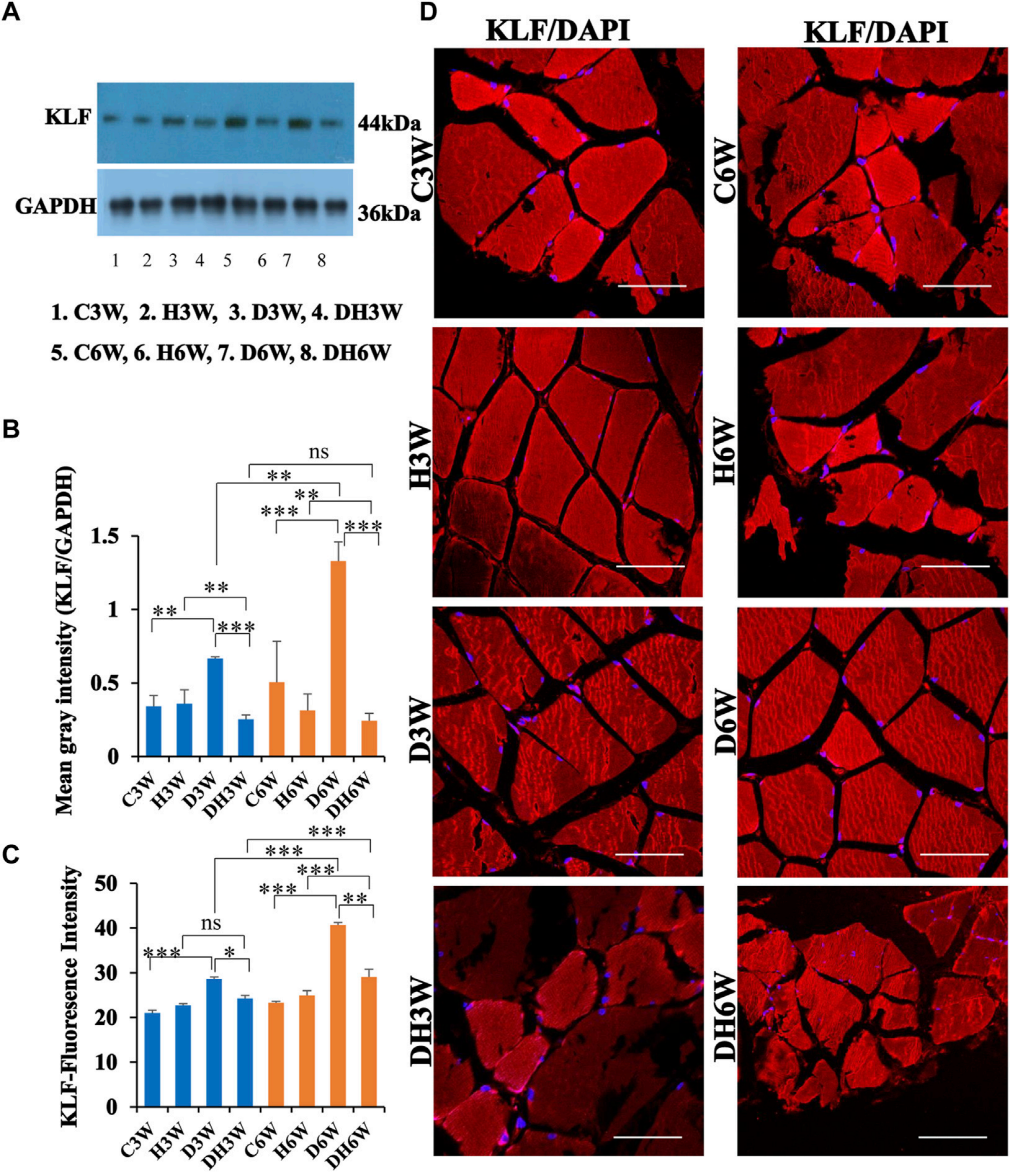
**(A,B)** Immunoblots probed with anti-KLF antibody **(A)** and the mean gray intensity of immunoblots **(B)**. Note the significantly increased expression of KLF (mean gray intensity) in D3W and D6W compared to C3W and C6W, respectively. KLF expression was decreased significantly in DH3W and DH6W compared to D3W and D6W respectively. There was a significant increase in KLF expression from short-term (D3W) to long-term (D6W). **(C,D)** KLF immunofluorescence intensity **(C)** and representative cross-section of muscle fibers stained for anti-KLF immunofluorescence in short-term (left panel) and long term (right panel) experiments **(D)**. Western blot data of KLF expression is further supported by fluorescence intensity measured on the anti-CD68 immunostained sections of the muscle fibers in most of the comparisons. Note large number of muscle fibers showing immunofluorescence in D3W, DH3W, D6W, and DH6W (*, *p* < 0.05; **, *p* < 0.01; ***, *p* < 0.001, One-way ANOVA, Bonferroni multiple comparison tests, n = 6 in all groups). Scale bar = 50 µm.

Western blot analysis for expression of KLF protein in the skeletal muscle fibers showed a significant increase in diabetic groups both in short-and long term experiments compared to control groups (C3W vs. D3W, *p* < 0.01; C6W vs. D6W, *p* < 0.001; One-way ANOVA, Bonferroni multiple comparison tests, n = 6 in all groups, Figures 8A,B). However, expression of KLF was decreased in diabetic rats subjected to heat therapy both in short- and long-term experiments compared to respective control groups (H3W vs. DH3W, *p* < 0.01; H6W vs. DH6W, *p* < 0.01). Expression of KLF was significantly decreased in diabetic rats subjected to heat therapy compared to diabetic rats, that are not subjected to heat therapy both in short- and long term experiments (D3W vs. DH3W, *p* < 0.001; D6W vs. DH6W, *p* < 0.001). There was a significant increase in the expression of KLF in long-term diabetic group compared to respective short-term diabetic group (D3W vs. D6W, *p* < 0.01); There was no significant difference between DH3W and DH6W (*p* > 0.05, Figures 8A,B). Western blot analysis data of KLF was supported by immunofluorescence intensity data measured on the cross sections of the muscle fibers stained for anti-KLF immunofluorescence both in short- and long term experiments (Figure 8C). The cross sections of the skeletal muscle, stained for anti-KLF immunofluorescence, showed many positive immunostained muscle fibers in diabetic rats (Figure 8D, 3^rd^ row) and their expression decreased in diabetic rats subjected to heat therapy (Figure 8D, 4^th^ row).

#### 3.6.3 MAFbx

**FIGURE 9:**
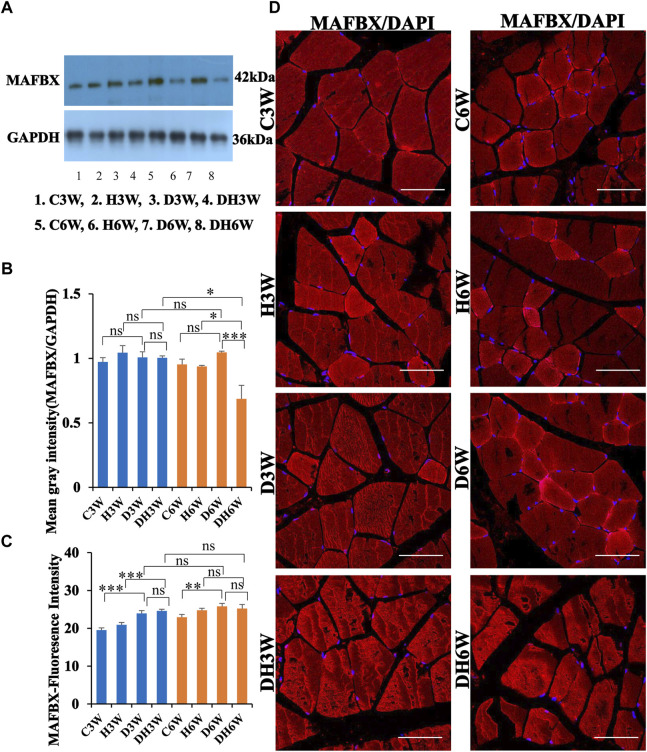
**(A,B)** Immunoblots probed with anti-MAFBX antibody **(A)** and mean gray intensity of immunoblots **(B)**. There were no significant differences in the expression of MAFBX (mean gray intensity) between groups in short term experiment. However, it was decreased significantly in DH6W compared to D6W and significantly less compared to DH3W. **(C,D)** MAFBX immunofluorescence intensity **(C)** and representative cross-section of muscle fibers stained for anti-MAFBX immunofluorescence in short-term (left panel) and long-term (right panel) experiments **(D)**. Immunofluorescence data showed significantly increased fluorescence intensity of MAFBX in D3W and D6W compared to C3W and C6W respectively. There was no significant difference in MAFBX expression from short-term (D3W and DH3W) to long-term (D6W and DH6W). Note large number of muscle fibers showing immunofluorescence in D3W, DH3W, D6W, and DH6W (*, *p* < 0.05; **, *p* < 0.01; ***, *p* < 0.001, One-way ANOVA, Bonferroni multiple comparison tests, *n* = 6 in all groups). Scale bar = 50 µm.

Western blot analysis for expression of MAFbx protein in the skeletal muscle fibers did not show any significant change in diabetic groups both in short-and long term experiments compared to control groups (C3W vs. D3W, *p* > 0.05; C6W vs. D6W, *p* > 0.05; One-way ANOVA, Bonferroni multiple comparison tests, n = 6 in all groups, Figures 9A,B). However, expression of MAFbx was decreased in diabetic rats subjected to heat therapy in long-term experiments, but not in short-term experiment, compared to respective control groups (H3W vs. DH3W, *p* > 0.05; H6W vs. DH6W, *p* < 0.05). Expression of MAFbx was significantly decreased in diabetic rats subjected to heat therapy compared to diabetic rats, that were not subjected to heat therapy in long-term experiments, but not in short-term experiments (D3W vs. DH3W, *p* > 0.05; D6W vs. DH6W, *p* < 0.001). There was no significant difference in the expression of MAFBX in long-term diabetic groups compared to respective short-term diabetic groups (D3W vs. D6W, *p* < 0.01); There was a significant decrease in expression of MAFbx between DH3W and DH6W (*p* < 0.05, Figures 8A,B). Immunofluorescence intensity data measured on the cross sections of the muscle fibers stained for anti-MAFbx immunofluorescence sowed a significant increase in diabetic rats both in short- and long-term experiments compared to control groups (C3W vs. D3W, *p* < 0.001; C6W vs. D6W, *p* < 0.01; Figure 8C) and diabetic and diabetic rats subjected to heat therapy for 3-weeks, but not in 6-weeks treatment group compared to respective control groups (H3W vs. DH3W, *p* < 0.001; H6W vs. DH6W, *p* > 0.05, Figure 8C). There was no significant difference in the fluorescence intensity when compared between diabetic and diabetic rats subjected to heat therapy both in short- and long-term experiments (D3W vs. DH3W, *p* > 0.05; D6W vs. DH6W, *p* > 0.05). Further, there were no significant changes in fluorescence intensity from 3-weeks to 6-weeks in diabetic and diabetic rats subjected to heat therapy (D3W vs. D6W, *p* > 0.05; DH3W vs. DH6W, *p* > 0.05). The cross sections of the skeletal muscle, stained for anti-MAFbx immunofluorescence, showed many positive immunostained muscle fibers in diabetic rats (Figure 9D, 3^rd^ row) and in diabetic rats subjected to heat therapy (Figure 9D, 4^th^ row).

In brief, heat therapy for diabetic rats for 3 weeks (short-term, DH3W) and 6 weeks (long-term, DH6W) significantly decreased the expression of degenerative muscle proteins, CD68 and KLF, but not MAFbx compared to diabetic rats that are not subjected to heat therapy (D3W and D6W).

### 3.7 Expression of muscle regenerative biomarkers (AKT, mTOR, and HSP70) in skeletal muscle tissue

#### 3.7.1 AKT

**FIGURE 10:**
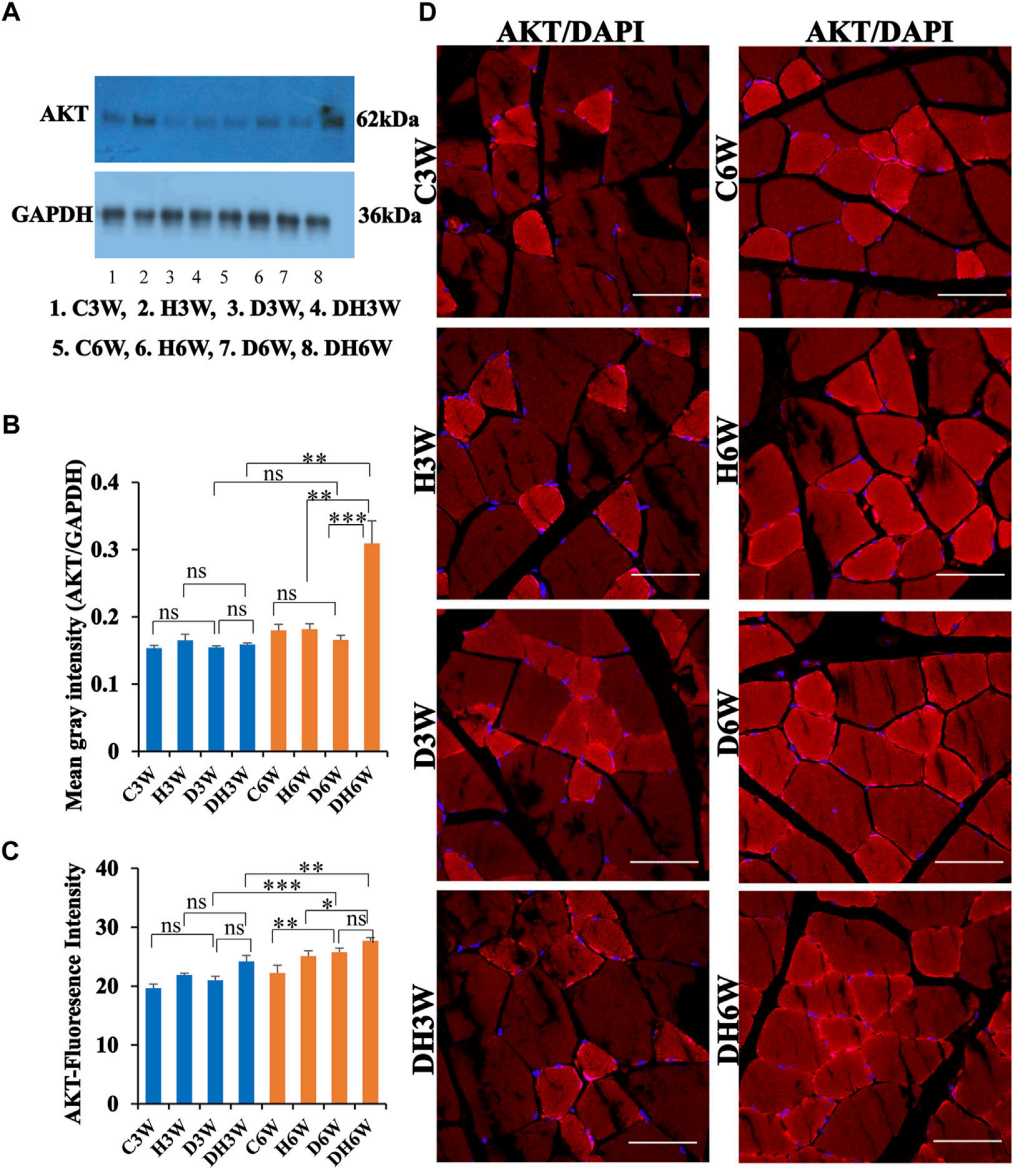
**(A,B)** Immunoblots probed with anti-AKT antibody **(A)** and mean gray intensity of immunoblots. There were no significant differences in the expression of AKT (mean gray intensity) between groups in short-term experiment. However, it was increased significantly in DH6W compared to H6W and significantly higher than D6W. There was a significant increase in AKT expression from short term (DH3W) to long term (DH6W). **(C,D)**-AKT immunofluorescence intensity **(C)** and representative cross-section of muscle fibers stained for anti-AKT immunofluorescence in short-term (left panel) and long-term (right panel) experiments **(D)**. Immunofluorescence data showed significantly increased fluorescence intensity of AKT in D6W and DH6W compared to C6W and H6W respectively. There was a significant increase in AKT immunofluorescence from short-term (D3W and DH3W) to long-term (D6W and DH6W). Note large number of muscle fibers showing immunofluorescence in D3W, DH3W, D6W, and DH6W (*, *p* < 0.05; **, *p* < 0.01; ***, *p* < 0.001, One-way ANOVA, Bonferroni multiple comparison tests, n = 6 in all groups). Scale bar = 50 µm.

Western blot analysis for expression of AKT protein in the skeletal muscle fibers did not show any significant change in diabetic groups both in short-and long-term experiments compared to control groups (C3W vs. D3W, *p* > 0.05; C6W vs. D6W, *p* > 0.05; One-way ANOVA, Bonferroni multiple comparison tests, *n* = 6 in all groups, Figures 10A,B). However, expression of AKT was increased in diabetic rats subjected to heat therapy in long term experiments, but not in short-term experiment, compared to respective control groups (H3W vs. DH3W, *p* > 0.05; H6W vs. DH6W, *p* < 0.001). There was no change in the expression of AKT in diabetic rats subjected to heat therapy compared to diabetic rats, that were not subjected to heat therapy in short-term experiments, but not in long-term experiments it was increased significantly (D3W vs. DH3W, *p* > 0.05; D6W vs. DH6W, *p* < 0.001). There was no significant difference in the expression of AKT in long term diabetic groups compared to respective short term diabetic groups (D3W vs. D6W, *p* < 0.01); There was a significant increase in expression of AKT between DH3W and DH6W (*p* < 0.05, Figures 10A,B). Immunofluorescence intensity data measured on the cross sections of the muscle fibers stained for anti-AKT immunofluorescence did not show any significant change in diabetic rats both in short- and long term experiments compared to control groups (C3W vs. D3W, *p* > 0.05; C6W vs. D6W, *p* > 0.05; Figure 10C) and diabetic rats subjected to heat therapy for 3-weeks, but not in 6-weeks treatment group compared to respective control groups (H3W vs. DH3W, *p* < 0.001; H6W vs. DH6W, *p* > 0.05, Figure 10C). There was no significant change in the fluorescence intensity when compared between diabetic and diabetic rats subjected to heat therapy in short- and long-term experiments (D3W vs. DH3W, *p* > 0.05; D6W vs. DH6W, *p* > 0.05). Further, there was a significant increase in fluorescence intensity from 3 weeks to 6 weeks in diabetic and diabetic rats subjected to heat therapy (D3W vs. D6W, *p* < 0.001; DH3W vs. DH6W, *p* < 0.01). The cross sections of the skeletal muscle, stained for anti-AKT immunofluorescence, showed a many positive immunostained muscle fibers in diabetic rats (Figure 10D, 3^rd^ row) and in diabetic rats subjected to heat therapy (Figure 10D, 4^th^ row).

#### 3.7.2 mTOR

**FIGURE 11:**
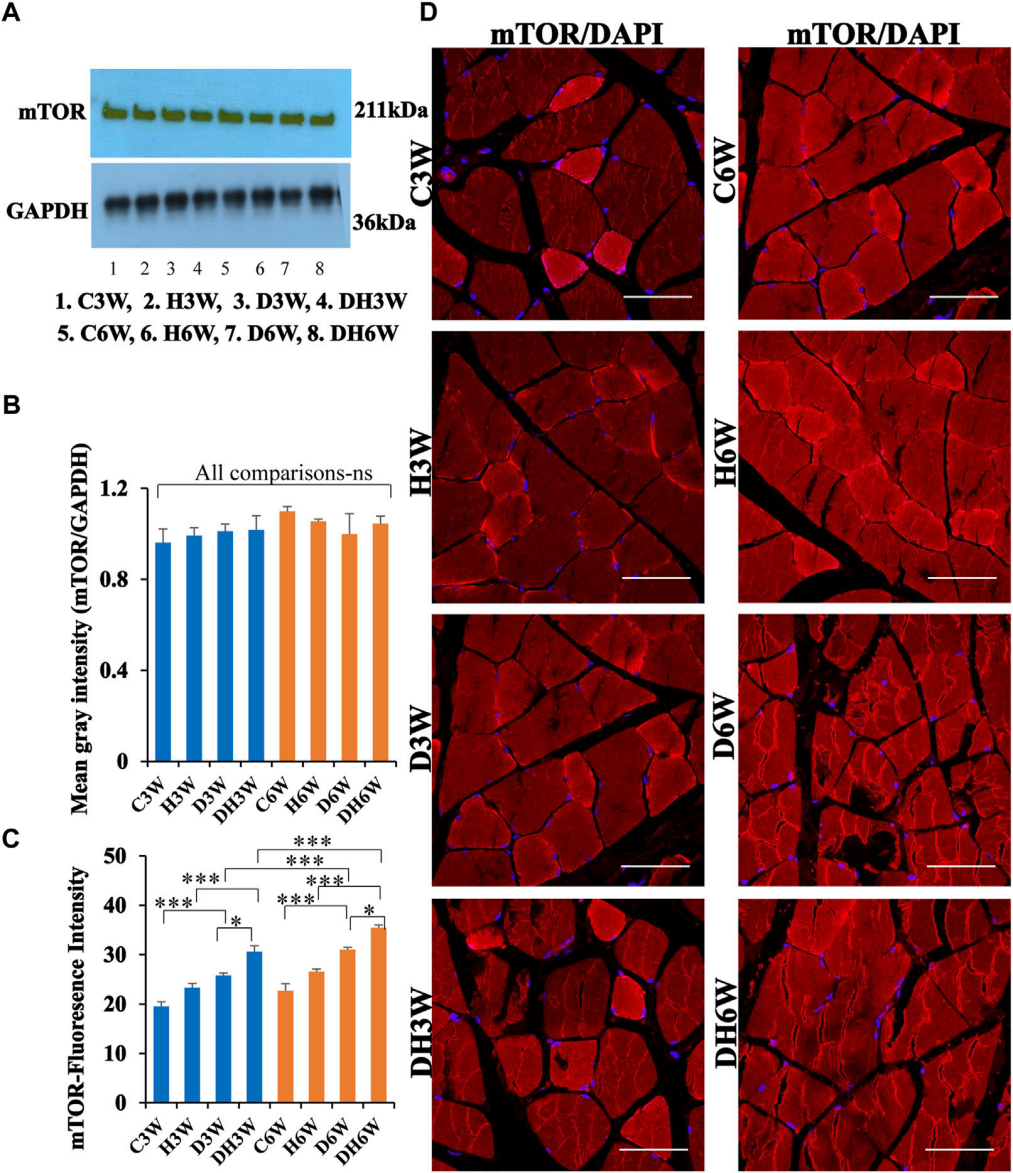
**(A,B)**-Immunoblots probed with anti-mTOR antibody **(A)** and mean gray intensity of immunoblots. Note there is no significant difference in expression of mTOR between groups in short- and long-term experiments. Note significantly increased immunofluorescence of mTOR in D3W and D6W compared to C3W and C6W respectively. **(C,D)**-mTOR immunofluorescence intensity **(C)** and representative cross-section of muscle fibers stained for anti-mTOR immunofluorescence in short-term (left panel) and long-term (right panel) experiments **(D)**. mTOR fluorescence was increased in DH3W and DH6W compared to D3W and D6W respectively. There was a significant increase in mTOR expression from short-term (D3W and DH3W) to long-term (D6W and DH6W). Note large number of muscle fibers showing immunofluorescence in D3W, DH3W, D6W, and DH6W. (*p < 0.05; ***, *p* < 0.001, One-way ANOVA, Bonferroni multiple comparison tests, n = 6 in all groups). Scale bar = 50 µm.

Western blot analysis for expression of mTOR protein in the skeletal muscle fibers did not show any significant change all groups in short-and long-term experiments compared to control groups (Figures 11A,B). Immunofluorescence intensity data measured on the cross sections of the muscle fibers stained for anti-mTOR immunofluorescence showed a significant increase in diabetic rats both in short- and long-term experiments compared to control groups (C3W vs. D3W, *p* < 0.001; C6W vs. D6W, *p* < 0.001; Figure 11C) and diabetic rats subjected to heat therapy in both short-term and long term experiments compared to respective control groups (H3W vs. DH3W, *p* < 0.001; H6W vs. DH6W, *p* < 0.05, Figure 11C). There was a significant increase in the fluorescence intensity when compared between diabetic and diabetic rats subjected to heat therapy in short- and long-term experiments (D3W vs. DH3W, *p* < 0.05; D6W vs. DH6W, *p* < 0.05). Further, there was a significant increase in fluorescence intensity from 3 weeks to 6 weeks in diabetic and diabetic rats subjected to heat therapy (D3W vs. D6W, *p* < 0.001; DH3W vs. DH6W, *p* < 0.001). The cross sections of the skeletal muscle, stained for anti-AKT immunofluorescence, showed many positive immunostained muscle fibers in diabetic rats (Figure 10D, 3^rd^ row) and in diabetic rats subjected to heat therapy (Figure 10D, 4^th^ row).

#### 3.7.3 HSP70

**FIGURE 12:**
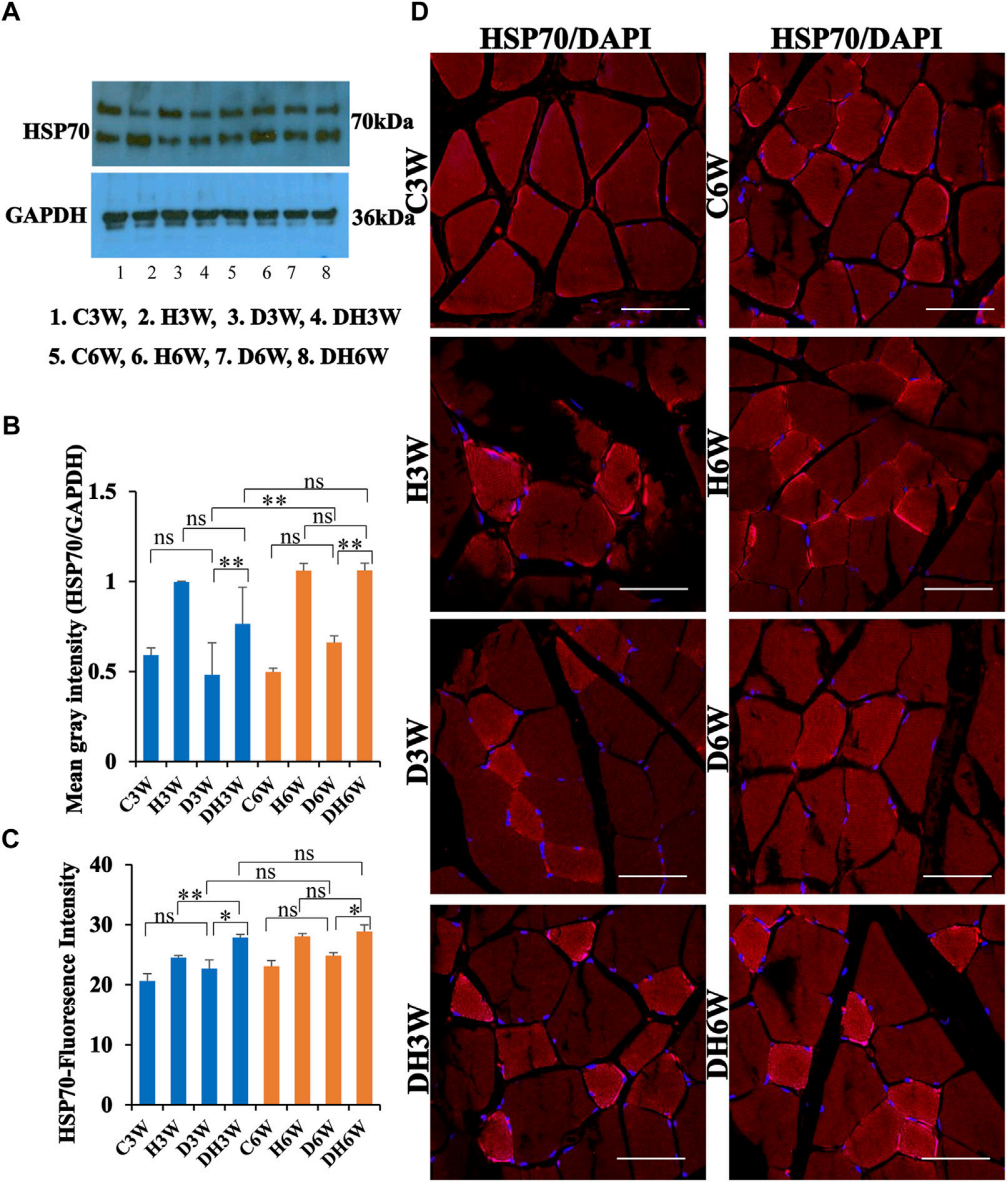
**(A,B)** Immunoblots probed with anti-HSP70 antibody **(A)** and mean gray intensity of immunoblots **(B)**. Note that there was no significant difference in the expression of HSP70 (mean grey intensity) in D3W and D6W compared to C3W and C6W respectively. HSP70 expression was significantly increased in DH3W and DH6W compared to D3W and D6W, respectively. There was a significant increase in HSP70 expression from short-term (D3W) to long-term (D6W). **(C,D)** HSP70 immunofluorescence intensity **(C)** and representative cross section of muscle fibers stained for anti-HSP70 immunofluorescence in short term (left panel) and long term (right panel) experiments **(D)**. Western blot data of HSP70 expression is further supported by fluorescence intensity measured on the anti-HSP70 immunostained sections of the muscle fibers in most of the comparisons. Note large number of muscle fibers showing immunofluorescence in D3W, DH3W, D6W, and DH6W (*p < 0.05, **, *p* < 0.01, One-way ANOVA, Bonferroni multiple comparison tests, *n* = 6 in all groups). Scale bar = 50 µm.

Western blot analysis for expression of HSP70 protein in the skeletal muscle fibers did not show any significant change in diabetic groups both in short-and long term experiments compared to control groups (C3W vs. D3W, *p* > 0.05; C6W vs. D6W, *p* > 0.05; One-way ANOVA, Bonferroni multiple comparison tests, *n* = 6 in all groups, Figures 12A,B). HSP70 protein expression in the skeletal muscle fibers increased significantly in diabetic rats subjected to heat therapy in short-and long-term experiments compared to control groups (D3W vs. DH3W, *p* < 0.01; D6W vs. DH6W, *p* < 0.01; Figures 12A,B. There was a significant increase in the expression of HSP70 in long term diabetic groups compared to respective short term diabetic groups (D3W vs. D6W, *p* < 0.01); There was no change in expression of HSP70 between DH3W and DH6W (*p* > 0.05, Figures 12A,B). Immunofluorescence intensity data measured on the cross sections of the muscle fibers stained for anti-HSP70 immunofluorescence did not show any significant change in diabetic rats both in short- and long-term experiments compared to control groups (C3W vs. D3W, *p* > 0.05; C6W vs. D6W, *p* > 0.05; Figure 12C). Diabetic rats subjected to heat therapy in both short-term and long-term experiments showed a significant increase in immunofluorescence compared to respective control groups (H3W vs. DH3W, *p* < 0.05; H6W vs. DH6W, *p* < 0.05, Figure 12C). Similarly, in the long-term experiment, there was a significant increase in the fluorescence intensity in diabetic rats subjected to heat therapy compared to diabetic rats (D6W vs. DH6W, *p* < 0.05). Further, there was no change in fluorescence intensity from 3 weeks to 6 weeks in diabetic and diabetic rats subjected to heat therapy (D3W vs. D6W, *p* > 0.05; DH3W vs. DH6W, *p* > 0.05). The cross sections of the skeletal muscle, stained for anti-HSP immunofluorescence, showed a large number of immunopositive muscle fibers in diabetic rats (Figure 12D, 3^rd^ row) and in diabetic rats subjected to heat therapy (Figure 12D, 4^th^ row).

In brief, short-term (DH3W) and long-term (DH6W) heat therapy on diabetic rats significantly increased the muscle regenerative proteins (AKT, mTOR, and HSP70) expression compared to the diabetic group D3W and D6W groups. Also, heat therapy increased the protein expression of these regenerative makers in the H3W and H6W groups.

## 4 Discussion

The present study investigated the effects of heat therapy on muscle strength, morphology (at light and electron microscopic levels) and expression of proteins that facilitate the degeneration and regeneration of skeletal muscles in STZ-induced diabetic rats. Results showed improved muscle strength (as revealed by neuromuscular functional tests), prevention of muscle atrophy (as revealed by body weight, quadriceps femoris muscle wet weight, light and electron microscopic observations of skeletal muscle structure), decreased and increased expression of degenerative and regenerative muscle proteins respectively (as revealed by western blot analysis and immunofluorescence intensity measurements) in STZ induced diabetic rats subjected to heat therapy either for three or six weeks compared to diabetic rats that are not subjected to heat therapy.

### 4.1 Heat therapy chamber temperature and core body temperature

The set 42°C temperature of the heat therapy chamber increased the core body temperature about 3°C above normal core body temperature in H and D + H groups. We measured the core temperature at the beginning of the session to ensure the core temperature was raised effectively and at the end to ensure it was maintained at the increased level and not shot up beyond 40°C. This increase in core temperature is required to bring about the expected changes (Hussain et al., 2018; Hannuksela et al., 2001; Beever et al., 2009; Sobajima et al., 2009) and is in fact tolerable to the rats as we did not see any adverse effects such as breathlessness, fainting, extreme fatigue. Previous studies which used a similar parameter also did not report any adverse effects (Chen et al., 1999; Naito et al., 2000; Sammut et al., 2001; Tamura et al., 2015; Sobajima et al., 2009). Sauna studies by Patrick and Johnson (2021) and many others indicate that humans can tolerate external temperatures of 80°C for more than 30 min and show positive outcomes. Since the increase in core temperature in the present study is within an acceptable range, data may be directly extrapolated to human subjects. However, they may need a fine adjustment in the temperature when applied to humans.

### 4.2 Blood glucose levels

In this study, STZ injection increased blood glucose levels within 48 h in injected rats (Figure 1). Their blood glucose level remained at the diabetic level(>200 mg/dl) throughout the experimental period suggesting a continuous effect of the diabetic condition on the body, muscles in particular. The constantly increased blood glucose level for a long duration (6 weeks) was consistent with previous studies, where diabetic status was induced and maintained for a long duration of 12 weeks. The blood glucose levels in 6 weeks diabetic rats in the present study is comparable to other studies (Afsari et al., 2008; Renno et al., 2008). A few studies have shown the beneficial effects of heat therapy on blood glucose levels (Hooper 1999; Kokura et al., 2007; Chung et al., 2008; Gupte et al., 2009; Bathaie, 2010; Kondo et al., 2012; Pallubinsky et al., 2020). Unlike these studies, in the present study, heat therapy on diabetic rats for both short- and long periods had no effect on the blood glucose levels. Studies such as ones performed by Gupte (2009) and Chung (2008) were focused on Type 2 diabetes and insulin resistance. They found benefits of heat therapy in terms of the elevating HSP70s levels, reducing diet/obesity induced hyperglycemia, and protection against insulin resistance through the action of inhibiting Jun NH2 terminal kinase and preventing its phosphorylation. Our present study proved the beneficial effects of heat therapy on muscle tissue in Type 1 diabetes mellitus (STZ to induce diabetes). Thus, heat therapy has beneficial effects regardless of the type of diabetes used for the study. The discrepancy with regard to heat therapy reducing the blood glucose in Gupte (2009) and Chung (2008) studies compared to our present study may be due to the different methods of heat therapy used, the duration of therapy since some therapies are provided for up to 12 weeks and type of diabetes (Gupte et al., 2009).

### 4.3 Body weight alterations

Results of the present experiment showed that non-diabetic control rats progressively gained body weight throughout the experimental period (Figure 2). Consistent with other studies, rats in diabetic groups showed gradual weight loss during the experimental period (Renno et al., 2008). However, in the present study, the weight loss in diabetic rats was drastic as rats who suffered from diabetes for 3 weeks showed a 24% decrease in body weight compared to controls, whereas the 6-week group showed approximately a 30% decrease in body weight compared to controls. Similar muscle mass loss was reported in diabetic patients (Reviewed by Nomura et al., 2018; Park et al., 2007). Interestingly, exposure of the diabetic rats to heat therapy for 6 weeks reduced the body weight loss to 25% suggesting a small (5%) but beneficial value of heat therapy in preserving body weight loss during the progression of diabetes, which may explain the enhanced muscle strength observed in these rats. Several studies have shown that heat therapy can reduce muscle mass loss in several conditions (Uehara et al., 2004; Goto et al., 2005; Kim et al., 2020a; Kim et al., 2020b).

### 4.4 Therapies used to prevent muscle atrophy

Heat therapy is one among the several therapies applied in preclinical experiments and clinics to slow down the muscle atrophy process (Goto et al., 2005; Ely et al., 2019). It has been shown that increasing the core body temperature and straining the body prevent muscular atrophy, allowing for a more natural acquisition of muscle mass, enhanced stress tolerance, higher insulin sensitivity, lower inflammation, and improved cardiovascular and circulatory functions (Patrick and Johnson 2021). Researchers have applied heat therapy through different modalities, such as diathermy and wet heat to prevent muscle atrophy (Hafen et al., 2019; Ohira et al., 2017). In diathermy, the heat was applied through short wave diathermy using a Megapulse-II physiotherapy machine, which transmits electromagnetic waves into the area of concern and causes an increase in kinetic energy within the tissue (Hafen et al., 2019). Even though shortwave diathermy on a rodent model provides consistent heat exposure, the dilemma remains in terms of reliability of heat exposure as the rodents can move during heat therapy while the heating tool used is stationary at one point. Wet heat or achieving heat using a water bath is another modality that has been studied in rodent models, which may be applied clinically. In wet heat therapy, heat stress was applied by immersing hindlimbs of the rats into a temperature-controlled water bath (Ohira et al., 2017). Nevertheless, the use of a water bath to induce hyperthermia provides complications of ensuring the full immersion of the rodents into the bath since rodents tend to explore the area and rise from the water level leaving only their hind limbs immersed. Although heating *via* a water bath is used, the lack of full body immersion and often the use of anesthesia to keep the rats in place (not to allow them to explore the area) may lead to inconsistent results.

The type of heat therapy applied in present study *via* full-body heating by keeping the rats in an enclosed heated chamber with a temperature set 4–5°C higher than body temperature has advantages such as no need for anaesthetizing, no movement restriction, and well-controlled environment (Uehara et al., 2004). The heat therapy protocol which we applied in the present study, is consistent with rodent studies (Naito et al., 2000; Tamura et al., 2015) and a sauna protocol for human subjects (heat therapy in a chamber maintained at 80°C, for 20–30 min, 4–7 times/week). In the sauna, skin temperature rises to 40°C and hence the core body temperature gets elevated significantly (∼39°C). As core temperature increases, cardiac output, heart rate increases as well. These physiological responses to the heat in saunas are very comparable to those felt during moderate-to strenuous activity. Therefore, using a sauna has been suggested as an alternative to exercising for those who are unable to do so owing to a chronic illness or physical restrictions (Patrick and Johnson, 2021).

### 4.5 Muscle strength analysis

In the present study, all three neuromuscular functional tests used (Kondziela’s inverted screen, rotarod, and extensor postural thrust tests) to assess the muscle strength in showed significantly improved muscle strength in diabetic rats subjected to heat therapy compared to diabetic rats that were not subjected to heat therapy for 3 or 6 weeks (Figure 3). The data collected through the above three motor functional tests establish the principal trend. While, diabetic untreated groups showed massive strength decreases due to the severe muscular atrophy they underwent due to the disease. The diabetic group treated with heat maintained their initial strength levels much more steadily. They had strength performance much closer to control and diabetic untreated groups.

The results of Kondziela’s inverted screen test in the present study are consistent with a previous study where mice exhibited severe motor function disabilities (20% decrease in latency to fall vs. 15% decrease in latency to fall in our study, Veniaminova et al., 2020). More significantly, by 6 weeks, diabetic rats showed a 71% decrease in latency to fall. Interestingly, heat therapy on the diabetic rats decreased the latency to fall to 14% at 6 weeks compared to the devastating 71% decrease in diabetic rats, showing positive and beneficial effects of heat therapy in maintaining proper motor function in diabetic rats.

In the present study, a 46% and 50% decrease in the fall time was recorded by diabetic rats in 3 weeks, and 6 weeks, respectively in the rotarod test, which is consistent with other studies where mice exhibited decreased motor function in the rotarod test after 3 weeks of diabetes, (Nagayach et al., 2014; Veniaminova et al., 2020). Muscle atrophy may be the main reason for the diabetic rats falling at a slower acceleration in both three and six week groups (Ishida et al., 2022). It may also be due to diabetes-induced neuropathy, which we have not studied. The increase in latency to fall is exacerbated in 6 weeks may be due to the increased muscle atrophy in them. However, the heat therapy diminished the decreased performance by 29% and 15% at three and 6-week periods respectively, suggesting a beneficial effect of heat therapy in maintaining muscle strength in diabetic rats studied. The decreased extensor postural thrust force (14% and 53%) in diabetic rats at 3 and 6 weeks respectively, are consistent with the results of the sciatic nerve crush injury study, where a 35% deficit was seen by 3 weeks and a 20% deficit by 6 weeks in the EPT test (Renno et al., 2016; Renno et al., 2021). The application of heat therapy diminished the destructive nature of diabetes for 6 weeks, and the deficit was reduced to a 32% decrease in force generation compared to normal controls.

### 4.6 Skeletal muscle atrophy analysis

In this study, we chose the quadriceps femoris muscle, for the skeletal muscle morphology study. Even though-diabetes induced muscle atrophy affects all muscles in the body, it has been suggested that extensor muscles atrophy more than flexor muscles (Gao et al., 2018). Keeping this in mind, we selected quadriceps femoris muscle for morphological study. Histomorphometry analysis of the quadriceps femoris muscle showed a significant decrease in the muscle fiber dimension both at the light and electron microscopic level in diabetic rats, which was counteracted by heat therapy (Figures 4–6). It is well known that diabetes mellitus is associated with loss of muscle mass, and strength (Appell 1990). In general, atrophying muscles continue to shrink in size, and they lose their previously functional or strength capability.

The results of the cross-sectional area of muscle fibers, a morphological parameter for muscle atrophy showed a remarkable progressive prevention of muscle atrophy by heat therapy. The cross sectional area of muscle fibers in diabetic untreated group (D3W) and diabetic heat therapy (DH3W) groups were 41.3% and 75.58% of control group (C3W) respectively by the end of the third week, accounting for 34.28% increase in cross sectional area of muscle fibers in DH3W groups. Similarly, 6 weeks of heat therapy resulted in 44.08% increase in cross sectional area of muscle fibers in DH6W groups (D6W-29.31% and DH6W 73.39% of control). Therefore, it can be confidently stated that heat therapy for the three and 6 weeks’ period has a beneficial role in preventing the muscle from atrophy. These significant reductions in muscle atrophy after three- and 6-week heat therapy may account for the enhanced muscle strength in those rats. A recent study revealed that a 7% decrease in the cross-sectional area of the vastus lateralis muscle during immobilization compared to control group and heat therapy reduced this CSA reduction to 4.5% (Hafen et al., 2019). In another study immobilization resulted in 10.8% decreased in muscle cross sectional area, and heat therapy brought down this reduction to 5.8% (Hirunsai and Srikuea, 2018). Comparing the current study with these studies it is patently show the beneficial effect of heat therapy in preventing muscle atrophy resulting from different conditions.

Electron microscopic observations on the longitudinal section of the quadriceps femoris muscle fibers showed decrease in the width of the muscle fibrils in the diabetic control groups at both the three- and six-week periods, which is in line with the diabetes-induced atrophy seen in previous studies (Fan et al., 2020; Wang et al., 2018). Guo et al. (2016) demonstrated hyperthermia at 39°C facilitated growth of sarcomere in myofibrils during myogenesis, associated up-regulation of several myofibril proteins. Liu et al. (2018) studied the ultrastructure of cardiac muscles and found the safety of extracorporeal cardiac shock wave therapy at microstructure, and ultrastructure levels. Furthermore, the diabetic groups showed a significantly smaller mitochondrion, an observation comparable to a previous report (Fan et al., 2020). However, in the diabetic group exposed to heat therapy the mitochondria are evidently larger and comparable to that in the control group. These findings favor the fact that heat therapy is beneficial for diabetic subjects as they allow those treated with heat at the specific protocol devised to maintain their initial levels of muscle mass, structure and diameter at the ultrastructural level. These electron microscopic findings support the hypothesis that heat therapy enhance the muscle structural properties, which may be the basis for enhanced performance in the motor functional tests, maintaining the quadriceps femoris muscle weight in diabetic rats exposed to heat therapy. Heat stress reported to increases the activity of the mitochondrial repertory chain complexes (complex I, II/III, IV, and V, Chen et al., 1999; Sammut et al., 2001). Mitochondrial respiratory chain protein content as well as enzyme activity is elevated under heat therapy in both rodents and human studies (Tamura et al., 2014; Hafen et al., 2019). Thus, increased size of mitochondria in D + H group in the present study may indirectly suggest an enhanced functional state of the mitochondria, however it needs to be investigated further with mitochondrial functional tests.

### 4.7 Analysis of muscle degenerative and regenerative markers

After knowing the functional and morphological benefit of heat therapy on skeletal muscle in diabetic rats and establishing the enhancement of muscle strength in diabetic rats by heat therapy for 3 weeks to a smaller extent and to a significant extent by 6-week, our novel study further investigated the biochemical mechanisms of muscle degeneration and regeneration that led to these changes by western blot and immunohistochemical analysis of muscle degenerative (CD68, KLF, and MAFBx) and regenerative (Akt, mTOR, and HSP70) markers.

### 4.8 Modulation of degenerative markers

#### 4.8.1 CD68

The increase in CD68 levels in the diabetic group (71% after 3 weeks, 95% after 6 weeks, Figures 7A–D) observed in our study is in line with previous studies where a significant increase (>75%) in CD68 expression levels in diabetic subjects was reported (Ehses et al., 2007; Sisino et al., 2013). CD68 quantity is reportedly increased in several conditions such as muscle damage in untrained exercise (Luk et al., 2021), muscular dystrophy (Howard et al., 2012), Placenta of diabetic rats (Sisino et al., 2013), in the islets of Langerhans in diabetic subjects (Ehses et al., 2007). Further, in this study, the enhanced expression of CD68 in diabetic rats was significantly reduced (47%) by heat therapy for three or 6 weeks. This substantiates that heat therapy is an effective technique to lower the extent of skeletal muscle atrophy in diabetes (Nonaka et al., 2015).

#### 4.8.2 KLF

The present study showed that heat therapy on diabetic rats reduces KLF protein expression and immunofluorescence intensity from 73% to 26% (Figures 8A–D), along with enhanced muscle strength. The KLF is a transcription factor that leads to diabetes-induced muscle loss when found in abundance (Liu et al., 2021). Regulators of this transcription factor are either through increased blood glucose levels or through WWP1 protein; both play crucial roles in regulating and slowing down the degradation of KLF15 protein (Hirata et al., 2019). In the skeletal muscles of diabetic animals, where muscle atrophy is seen, KLF was reported to be in abundance suggesting the role of KLF in muscle atrophy in diabetes (Hirata et al., 2019). The role of KLF in muscle atrophy was further proved in KLF-15 knock-out mice, which were resistant to diabetes-induced skeletal muscle mass decline (Hirata et al., 2019). Under normal circumstances, WWP1 promotes the breakdown of KLF15 protein by attaching ubiquitin to it, resulting in a low level of cellular KLF15. During diabetes, KLF is increased in the muscle through the Krebs cycle, which is activated during diabetes which results in the degradation and atrophy of muscle tissue (Pollak et al., 2018), which is counteracted by heat therapy. This abundance in KLF15, in turn, increases the rate of diabetes-induced muscle atrophy through its ability to affect downstream target genes and code for protein catabolism (Gray et al., 2007).

#### 4.8.3 MAFbx or Atrogin 1

The present study showed that MAFbx significantly increased in rats who suffered from diabetes for 6 weeks, and heat therapy during a 6-week diabetic period decreased the activity of MAFbx and downregulated muscle atrophy (as evidenced by increased muscle strength, Figures 9A–D). Several studies are in support of our results. MAFBX is a muscle-specific ubiquitin ligase, that has a role in cases of muscle atrophy seen in several disease conditions (Bonaldo and Sandri, 2013). Studies have shown increased MAFBX in muscle atrophy associated with denervation and type-1 diabetes (Zhao et al., 2008; Ohira et al., 2017). Increase in MAFBX (Atrogin-1) promotes muscle atrophy by enhancing the degradation of MyoD (a key muscle transcription factor) and eIF3f (an essential activator of protein synthesis) thereby facilitating decreased protein synthesis in muscle leading to muscle atrophy (Bonaldo and Sandri, 2013; Sanchez et al., 2013). It has been shown that in a denervated muscle which were undergoing atrophy, transcription of MAFbx and *MuRF-1*(muscle RING-finger protein-1) genes and ubiquitination of proteins was upregulated, (Ohira et al., 2017). It has been claimed that enhancement of the ubiquitin ligases such as muscle atrophy F box/atrogin-1 (MAFbx) and muscle RING finger 1 (MuRF1) results in muscle wasting and atrophy in diabetic experimental animals (Zhao et al., 2008). During muscle disuse atrophy, upregulation of genes forming ligases such as MAFbx has been reported (Liu et al., 2021). The beneficial effect of heat therapy observed in our study is supported by a few studies. The heat stress therapy (42°C for 30 min) applied on alternate days for 2 weeks inhibited the upregulation of *Atrogin-1* and attenuated atrophy in denervated muscles (Ohira et al., 2017).

### 4.9 Modulation of regenerative markers

#### 4.9.1 Akt

Akt, otherwise known as protein kinase B, acts in conjunction with mTOR. Results of present study revealed a significant decrease in Akt levels in 6-week diabetic rats (Figures 10A–D). This observation agrees with a human study where in a diabetic patient, the activity of Akt kinase in response to insulin was reported to reduce by 34% compared to healthy controls (Krook et al., 1998). It is well known that the primary regulator of protein synthesis in skeletal muscle is the activation of the mammalian target of rapamycin (mTOR). Akt activates mTOR in turn, along with insulin. The decreased Akt activity, which can no longer can activate mTOR, leads to a substantial reduction of protein synthesis in skeletal muscles (Perry et al., 2016). Thus, in the present study, the observed decrease in Akt level in 6-weeks diabetic rats might have caused muscle atrophy. The present study showed a significant increase in Akt levels in 6-week diabetic rats subjected to heat therapy. The increase in Akt levels correlates to decreased muscle atrophy (as evidenced by increased muscle strength), which is in line with studies where heat stress, increased levels of phosphorylated Akt and HSP70 (Ohira et al., 2017). Phosphorylated Akt and 70-kDa ribosomal protein S6 kinase (p70S6K) were shown to be significantly increased in rat leg muscles (soleus and plantaris) after hindlimb immersion into the water at 41°C for 30 min (Yoshihara et al., 2013). Thus, it may be proposed that heat stress may serve as an effective therapeutic approach to ameliorate the reduction in muscle mass. An increase in Akt level (26%) and increased muscle strength observed in our study is further supported by studies relating the function of Akt and insulin in muscle metabolism through glucose transport and protein synthesis. It has been reported that through the actions of Akt-mediated mTORC1 activation, insulin regulates several downstream proteins *via* phosphorylation of Akt to increase protein synthesis, suggesting the role of Akt in enhancing the muscle mass (Huang et al., 2018). The importance of Akt not only lies with its activation of mTOR, but also *via* is phosphorolation of FOXO, the transcription factors regulating the ubiquitin ligases responsible for protien degradation (Brunet, 1999). This phosphorolation holds FOXO in the cytosol and does not allow it to transcribe E3 ligases.

#### 4.9.2 mTOR

The mTOR is a primary regulator of protein synthesis in skeletal muscle (Bodine et al., 2001; Watson and Barr, 2014). mTOR acts in conjunction with Akt protein. In cases where Akt is hypoactive and no longer can activate mTOR, a significant reduction in protein synthesis happens in skeletal muscles. The results of our study did not show any significant difference between groups’ mTOR protein levels, even though its fluorescence intensity increased in diabetic rats and further increased in diabetic rats subjected to heat therapy (Figures 11A–D). The absence of difference between groups in mTOR protein level could be explained by aberrantly increase in mTOR in diabetes or metabolically stressed conditions (Mao and Zhang 2018). Therefore, an increase in mTOR by heat therapy may be nullified by its aberrant increase in diabetic rats. In studies where obese db/db mice show muscle atrophy, there is a 43% increase in the rate of protein degradation compared to lean controls (Perry et al., 2016). Moreover, HSP90, a heat shock protein upregulated in heating events, binds directly to Akt to modulate its activity. When HSP90 is inhibited, the dephosphorylation of Akt occurs; thus, it is inactive and cannot activate mTOR. Nevertheless, mTOR is known as one of the main culprits in signaling for hypertrophy. The Akt/mTOR pathway is a vital mechanism in muscular hypertrophy, capable of preventing atrophy *in vivo*. Although Verges (2017) reported the importance of mTOR and the mTOR pathway as vital to signaling growth and regulation of glucose and lipid metabolism (Verges 2017), Mao and Zhang showed that mTORC1 activity exerts a significant effect on muscle mass by affecting the autophagy process. Sustained activation of mTORC1 in skeletal muscle reduces muscle mass and muscle fiber size in old mice, leading to a late-onset myopathy tightly associated with other metabolic pathways (Mao and Zhang 2018). Thus, it is apparent that mTOR involvement in the muscle is highly complex.

#### 4.9.3 HSP70

The HSP70 protein has several functional roles in the cells including protein structural modifications and chaperoning. The present study showed significantly increased expression of HSP70 in 6-week diabetic rats that are subjected to heat therapy (Figures 12A–D). It has been reported that applying heat on immobilized muscle during heat therapy significantly increases HSP70 and HSP90 and prevents the loss of proteins associated with mitochondrial respiratory complexes, in addition to increasing the intramuscular temperature (Hafen et al., 2019). The overexpression of HSP70 has been shown to reduce the immobilization-induced atrophy in rat skeletal muscle by ∼75%. The prevention of muscle atrophy by increased HSP70 levels in skeletal muscle during heat therapy is said to be by limiting the increase in atrogin-1/MAFbx and MuRF-1, which are responsible for muscle atrophy (Hafen et al., 2019). HSP70 was increased in diabetic rats in the present study. Since diabetes and its concomitant oxidative stress are forms of stress (Nakhjavani et al., 2010), it is no surprise that a heat shock response was noted in them. The body activates HSPs to counteract the effects of the diabetic stress it encounters (Nakhjavani et al., 2010). Moreover, the increase in HSP70 expression level is consistent with that of previous studies, wherein HSP70 levels increased by 51% (Hafen et al., 2019). This substantiates that daily heat therapy increases HSP70 levels significantly and serves to protect skeletal muscle from degradation and attenuate diabetic-induced muscle atrophy.

## 5 Conclusion

Previous studies have shown the effectiveness of heat therapy on physical performance and how it acts at the molecular level to boost regenerative muscle markers as a mechanism of their action on immobilization-induced or disuse atrophy. This study aimed to find whether or not heat therapy can attenuate muscle atrophy in diabetic rats for short and long periods. Data showed a significant improvement in performance of the diabetic rats exposed to heat therapy. The heat therapy on diabetic rats demonstrated increased muscle strength during neuromuscular functional tests and increase in muscle fiber cross-sectional area, sarcomere length and wet weight of the muscle studied. In addition, it improved the regenerative protein markers and decreased the degenerative protein markers. Based on the results of the present study, it can be authoritatively stated that heat therapy attenuates muscle atrophy in the animal diabetic model. This novel animal study conclusively favors the use of heat-therapy on diabetic human subjects as a viable remedy to aid in curbing muscle atrophy and reinforcing overall health. Torres et al., 2004; Clijsen et al., 2022.

## Data Availability

The original contributions presented in the study are included in the article/Supplementary Material, further inquiries can be directed to the corresponding author.
