# Supplementary figures and images for: The impact of heat therapy on neuromuscular function and muscle atrophy in diabetic rats
Source: Front Physiol. 2023 Jan 5;13:1039588. doi: 10.3389/fphys.2022.1039588 (PMC9849254; doi:10.3389/fphys.2022.1039588)

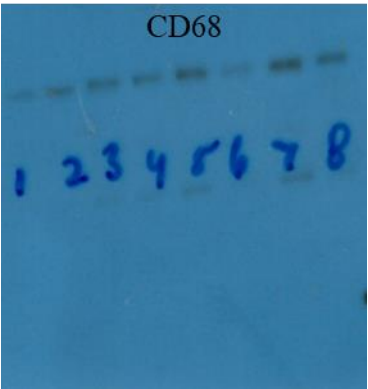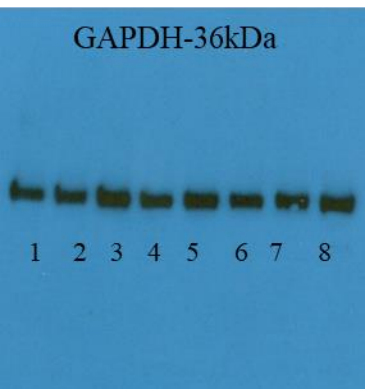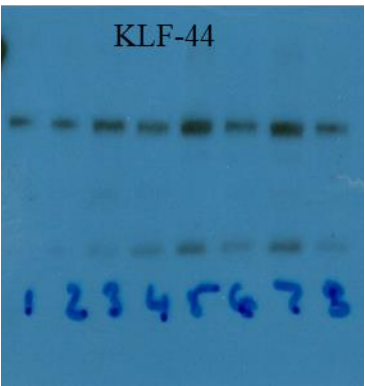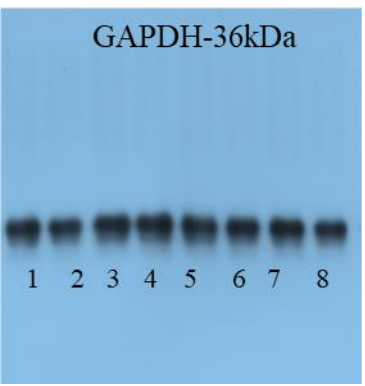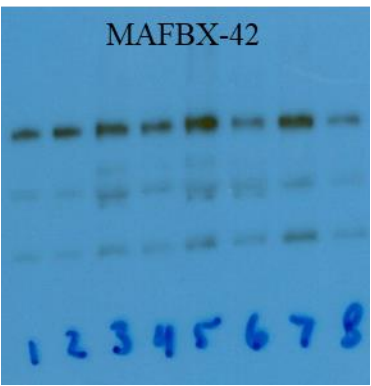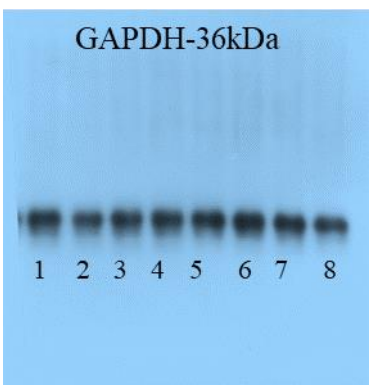

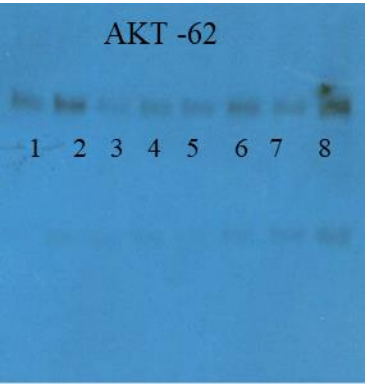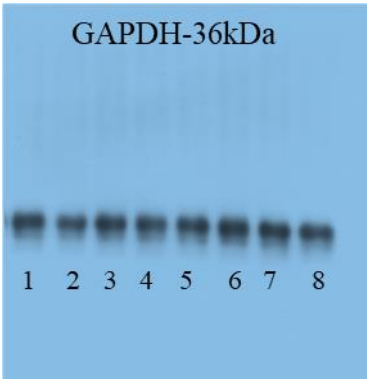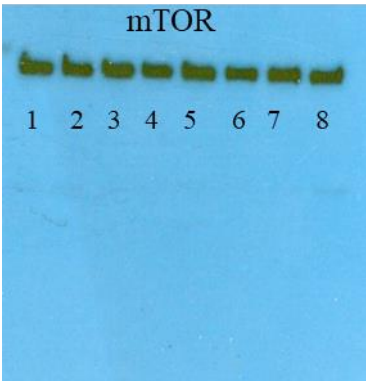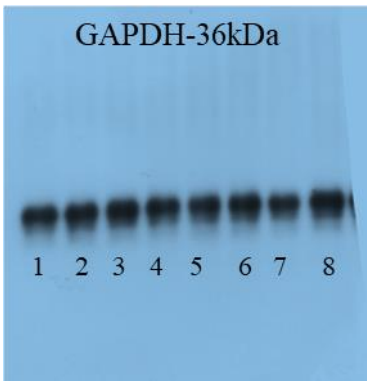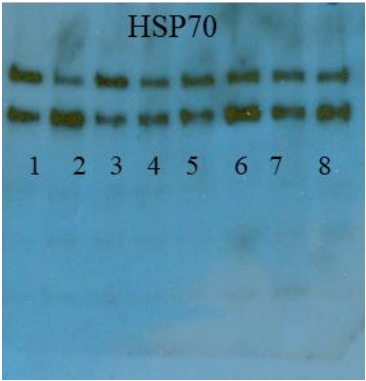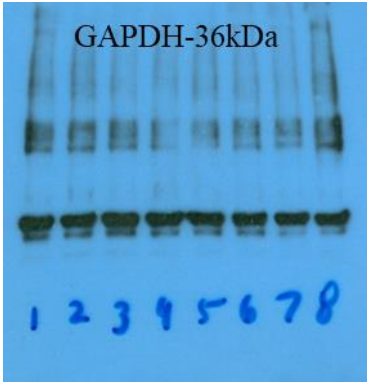

Supplement: Supplementary file 1 [file DataSheet1.PDF]
